# Perceptions of personal and public risk: Dissociable effects on behavior and well-being

**DOI:** 10.1007/s11166-022-09373-0

**Published:** 2022-04-02

**Authors:** Laura K. Globig, Bastien Blain, Tali Sharot

**Affiliations:** 1 Affective Brain Lab, Department of Experimental Psychology, University College London, London, UK; 2 The Max Planck UCL Centre for Computational Psychiatry and Ageing Research, University College London, London, GB UK

**Keywords:** Pandemic, Decision-making, Risk perception, Well-being, Affect, Behavior, I – Health, Education and Welfare, I – Health, Education and Welfare

## Abstract

When faced with a global threat peoples’ perception of risk guides their response. When danger is to the self as well as to others two risk estimates are generated—to the self and to others. Here, we set out to examine how people’s perceptions of health risk to the self and others are related to their psychological well-being and behavioral response. To that end, we surveyed a large representative sample of Americans facing the COVID-19 pandemic at two times (N_1_ = 1145, N_2_ = 683). We found that people perceived their own risk to be relatively low, while estimating the risk to others as relatively high. These risk estimates were differentially associated with psychological well-being and behavior. In particular, perceived personal but not public risk was associated with people’s happiness, while both were predictive of anxiety. In contrast, the tendency to engage in protective behaviors were predicted by peoples’ estimated risk to the population, but not to themselves. This raises the possibility that people were predominantly engaging in protective behaviors for the benefit of others. The findings can inform public policy aimed at protecting people’s psychological well-being and physical health during global threats.

**Supplementary information:**

The online version contains supplementary material available at 10.1007/s11166-022-09373-0.

When faced with a global threat people likely generate an estimate of the risk to oneself and to others. These estimates may diverge. In particular, it has been shown that people provide lower risk estimates to themselves than to others (Kuzmanovic et al., 2015). The divergence between personal risk perception and public risk perception raises a critical question. Namely, how do these diverging estimates relate to people’s psychological well-being and behavior. It has been suggested that low perception of personal risk is related to happiness (Dember & Penwell, 1980) and inversely related to depression and anxiety (Sharot, 2011; Strunk et al., 2006). However, if people perceive personal risk as relatively low but public risk as high it begs the question of how the two would act together. Would the respective effects cancel each other out, or would one factor dominate the other? With regards to behavior, a person’s estimates of vulnerability and future prospects will guide decision-making (Krieger et al., 2016). For example, underestimating risk of disease leads to reduced medical screenings (Krieger et al., 2016). Yet, a person’s predictions about the vulnerability of society may too drive behavior, especially in cases when a person’s own behavior can help protect others.

Second, if public policy is to be put in place to influence perceptions of risk, it is important to identify factors that may affect risk perception. Here, we hypothesized that perceived risk is related to a person’s sense of control. People tend to feel more optimistic about things they believe they can control (Harris, 1996; Helweg-Larsen & Shepperd, 2001; Klein & Helweg-Larsen, 2002; Zakay, 1984). While this sense of control is often overestimated (Langer, 1975; Tobias-Webb et al., 2017), the general belief is rational, because when outcomes are controllable risk will indeed be lower if a person takes action to avoid that risk. A strong sense of control may be linked exclusively to personal risk or it may be linked to both personal and public risk. The reasoning for the latter is that people with a high sense of control may be more likely to believe that outcomes in general are controllable, not just for themselves, but for others too. This may then lead to the conclusion that public risk is low as long as people take mitigating action.

To examine how perception of personal and public health risk relate to psychological well-being and behavior we surveyed people’s perception of the danger COVID-19 posed to them and to their fellow citizens during the 2020 global pandemic of the novel coronavirus. As the pandemic touched most of humanity it made it possible to examine the questions outlined above across a large and diverse population. To that end, we surveyed a representative sample of 1145 Americans across 30 States during March 26–29, 2020, as stay at home orders were being issued across the US (time 1). We measured personal and public risk perception, psychological well-being, compliance with behavioral recommendation to mitigate risk and people’s sense of control.

We then surveyed 683 participants again a month later while all states were under lockdown (time 2 – April 23–25, 2020). This allowed us to examine how risk perception, psychological well-being and behavior changed over the month. In line with research showing successful adaptation to hardships (Bonanno et al., 2002, 2004; Brickman et al., 1978; Dijkers, 1997; Frederick & Loewenstein, 1999) we predicted that psychological well-being would improve.

Understanding the relationship between COVID-19 risk perception, well-being and behavior is particularly important as governments worldwide were having to strike a balance between ensuring behavioral compliance with behavioral regulations to slow the spread of the virus (e.g., social-distancing, frequent handwashing) and maintaining citizens’ well-being during a time of uncertainty. Thus, knowledge of the relationship between risk perception, affect and behavior may aid in effective policymaking.

## Methods

### Participants, time 1

**Fig. 1:**
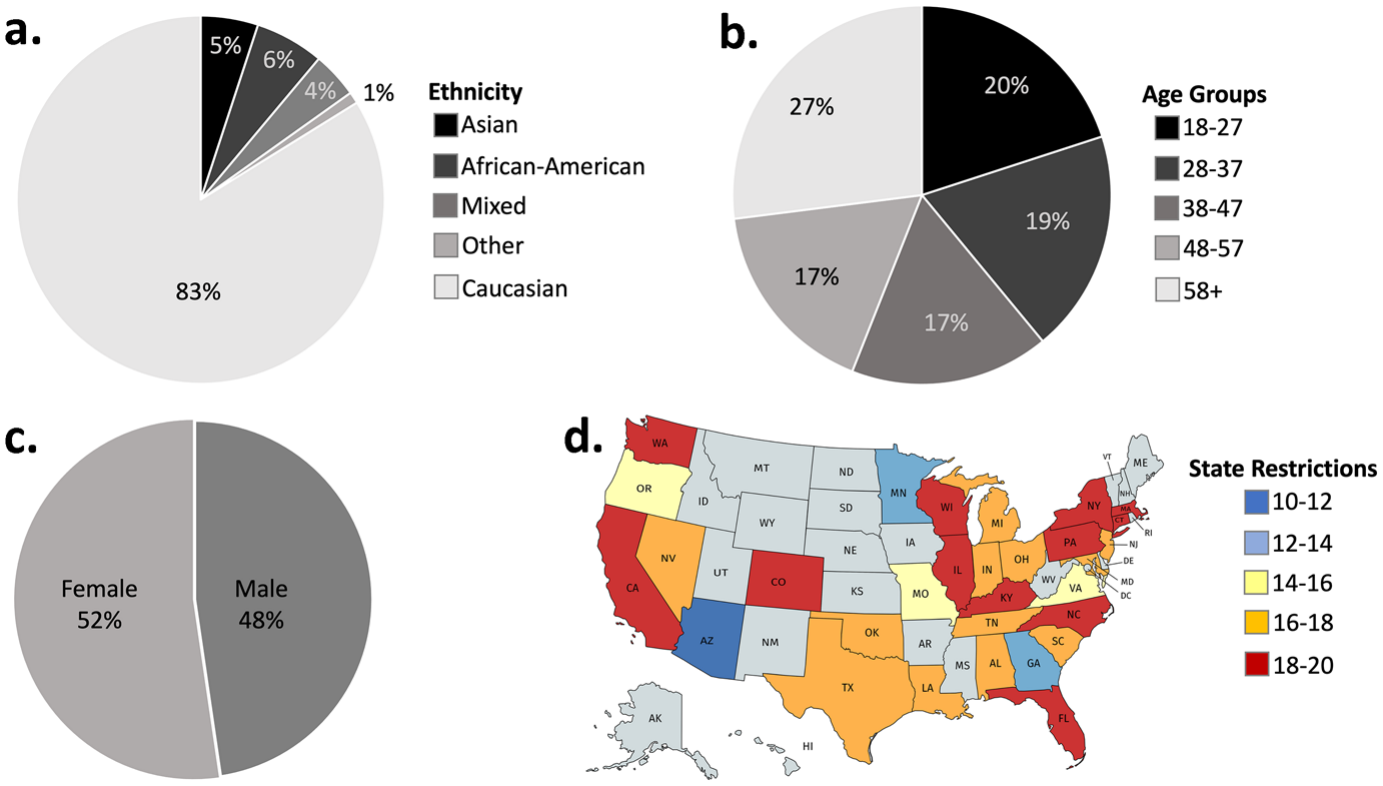
**Overview of demographics.** Sample (N = 1145) is representative of the US population in terms of (**a**) Ethnicity, (**b**) Age, and (**c**) Gender. (**d**) Participants resided in 30 US States at time of testing. Approximately 40 participants were tested in each of the states. We computed the level of each of those state’s behavioral restrictions using information available from the OxCGRT (Hale et al., 2020) (see Sect. 1) from 10–12 (most lenient in blue) to 18–20 (most severe in red). The demographics of participants in time 2 are similar to those portrayed here (see Sect. 1)

We tested 1166 individuals between March 26–29, 2020, representative of the US population in terms of age, gender and ethnicity (see Fig. 1a-c). The individuals were residing in 30 US states at the time of testing (Fig. 1d). They completed an online questionnaire on Prolific Academic. We tested participants’ engagement and attention by asking them to select a particular answer to various “catch trials” throughout the Survey (for example: *Please select ‘strongly disagree’*). Participants who did not select the required response more than once were excluded from analysis (N = 21). Thus, data of 1145 participants were analyzed (mean age = 44.00, SD = 15.59; females = 52.3%, Democrats = 69%, Republicans = 31%). 144 participants did not indicate ethnicity and could thus not be included in any models in which ethnicity was a factor. Participants provided informed consent and received $4.66 for their participation. Ethical approval was provided by the Research Ethics Committee at University College London.

### Participants, time 2

683 participants in time 1 completed a follow-up survey a month later (April 23–25, 2020) on Prolific Academic (mean age = 46.00, *SD* = 15.22; 18–27 years old = 13.5%; 28–37 years old = 17.9%; 38–47 years old = 18.2%; 48–58 years old = 18.4%; over 58 years old = 32.1%; females = 51.1%; Democrats = 68.4%; Republicans = 31.6%; Asian = 4.5%; African-American = 6.3%; Mixed = 4.5%; Other = 1.6; Caucasian = 83%). The individuals were residing in 30 states at the time of testing. None of the participants failed more than 1 of the catch trials. Participants provided informed consent and received $2.34 for their participation. Ethical approval was provided by the Research Ethics Committee at University College London.

### Materials, time 1

Participants completed an online survey which lasted approximately 30 min. In addition to the questions that formed this study, additional information was gathered as part of parallel studies not reported here. These focused mostly on habits, personality, psychopathology and other opinions regarding the crisis. We detail the additional information gathered in the Online Appendix. Below we detail the information gathered via the online survey which is part of the current study.

#### **Converting responses**:

In compliance with the policy of the Journal of Risk and Uncertainty all scores were converted to either 0 or 1 as detailed below. This is because responses were given on an ordinal scale and thus do not necessarily have cardinal or quantitative significance. Note that in cases where response were given on a 1–5 scale responses were converted to 0/1/2 as detailed below.

#### **Demographics**: 

Participants indicated their age, gender, ethnicity, level of education, household income, health insurance satisfaction, political orientation, whether they had children and their current place of residence. In compliance with the policy of the Journal of Risk and Uncertainty ordinal health insurance satisfaction ratings were converted as follows: Scores below the midpoint of the scale (< 3) were converted to 0 and those equal or above the midpoint (> = 3) were converted to 1.

### State restrictions

Using information about containment and closure policies available from the OxCGRT (Hale et al., 2020) we quantified the level of restrictions in each participant’s state on the date they completed the survey. In particular, we used eight policy indicators: 1) school closure (0 = no, 1 = recommend closing, 2 = require closing of some levels, 3 = require closing all levels), 2) workplace closure (0 = no, 1 = recommend closing, 2 = require closing of some sectors, 3 = require closing for all but essential workplaces), 3) cancellation of public events (0 = no, 1 = recommend cancelling, 2 = require cancelling), 4) restrictions on gatherings (0 = no, 1 = restrictions on very large gatherings > 1000 people, 2 = restrictions on gatherings between 101–1000 people, 3 = restrictions on gatherings between 11–100 people, 4 = restrictions on gathering of 10 people or less), 5) closure of public transport (0 = no, 1 = recommend closing, 2 = require closing), 6) “Stay at Home” requirements (0 = no, 1 = recommend not leaving the house, 2 = require not leaving house with exceptions for daily exercise, grocery shopping and essential trips, 3 = require not leaving house with minimal exceptions), 7) Restrictions on internal (0 = no measures, 1 = recommend not to travel between regions/cities, 2 = internal movement restrictions in place) 8) International travel controls issued movements (0 = no, 1 = screening arrivals, 2 = quarantine arrivals for some or all regions, 3 = ban arrivals from some regions, 4 = ban on all regions or total border closure). The sum level of restrictions was computed per state. In compliance with the policy of the Journal of Risk and Uncertainty scores were converted such that scores below the midpoint of the scale (< 12) were converted to 0 and those equal or above (> = 12) were converted to 1.

### Perceived relative personal risk

Participants were asked to indicate: “Relative to others of your age and gender do you think you are less/more likely to get COVID-19?” on a scale from 1 (much less likely) to 5 (much more likely). In compliance with the policy of the Journal of Risk and Uncertainty ordinal scores were converted as follows: 1 (much less likely) and 2 (less likely) were converted to 0; 3 (just as likely) was converted to 1, and 4 (more likely) and 5 (much more likely) were converted to 2.

### Perceived public risk

 Participants were asked to indicate: “Do you think COVID-19 presents a real danger to the health of the human population?” using continuous visual analogue scale from 0 (not really) to 100 (extreme danger). High numbers indicate high perceived public risk. In compliance with the policy of the Journal of Risk and Uncertainty ordinal scores were converted as follows: scores below the midpoint of the scale (< 50) were converted to 0 and those above (> = 50) were converted to 1.

### Happiness

Participants were asked two question to assess their happiness: (i) “Taken all together, how happy are you with your life these days? Mark your rating relative to the least and most happy time of your life.” Participants were asked to respond on a continuous visual analogue scale ranging from 0 (least happy time of your life) to 100 (most happy time of your life). (ii) “Think about right now. How happy are you at this moment?”. Participants were asked to respond on a continuous visual analogue scale ranging from 0 (very unhappy) to 100 (very happy). Results are the same regardless of which question we look at. Thus, we report results of the first question here and of the second in the Online Appendix. In compliance with the policy of the Journal of Risk and Uncertainty ordinal scores were converted as follows: scores below the midpoint of the scale (< 50) were converted to 0 and those equal or above (> = 50) were converted to 1.

### Anxiety

We assessed general anxiety using the short version of the State-Trait Anxiety Inventory (STAI, Marteau & Bekker, 1992). In compliance with the policy of the Journal of Risk and Uncertainty ordinal scores were converted as follows: scores below the threshold of clinical anxiety (< 40, Spielberger, 2010) were converted to 0, and scores equal or above the threshold of clinical anxiety (> = 40) were converted to 1.

### Behavioral compliance measures

Participants rated on a continuous visual analogue scale ranging from 0 (none at all) to 100 (a lot): a) “How much effort do you make to wash your hands regularly?”; b) “How much effort do you make to socially distance yourself from others?”; c) “How much effort do you make to avoid touching your face?”. On a continuous visual analogue scale ranging from 0 (zero) to 100 (many times) they also rated: d) “In the past week how many times have you been to another person's house?”; e) How many days this week have you been closer than 1 m to another person (except those you live with)?”. Items d) and e) were reverse coded to compute a mean score of behavioral compliance. To quantify behavioral compliance, we averaged each participant’s scores on all behavioral compliance questions and in compliance with the policy of the Journal of Risk and Uncertainty ordinal scores were converted as follows: scores below the midpoint of the scale (< 50) were converted to 0 and those above (> = 50) were converted to 1.

### Sense of control

Participants completed a questionnaire assessing sense of control (Lachman & Weaver, 1998). The questionnaire comprises of two sub-scales; personal mastery and perceived constraints. The personal mastery sub-score quantifies a person’s sense of efficacy or effectiveness in carrying out goals. The perceived constraints sub-score quantifies to what extent a person believes there are obstacles or factors beyond one’s control that interfere with reaching goals. To get one score reflecting sense of control we first inversed the score on the second scale, such that a higher score reflects less perceived constraints. We then averaged the two sub-scores such that a higher score reflects a higher sense of control. In compliance with the policy of the Journal of Risk and Uncertainty ordinal scores were converted as follows: scores below the midpoint of the scale (< 21) were converted to 0 and those above (> = 21) were converted to 1.

### Materials, time 2

Materials and procedure were the same as in time 1 except for the following changes**:**

1. In time 1 we assessed perceived relative personal risk by asking participants how likely they thought they were to get COVID-19 relative to others their age and gender. By contrast, perceived public risk was assessed by asking participants to indicate whether the virus presented a danger to the health of the human population. To rule out that the findings in time 1 were a result of assessing perceptions of “infection risk” vs. “danger” rather than assessing perception related to the self vs. the human population, we changed the question assessing “perceived public risk”. In particular we asked participants “How likely do you think a person is to get COVID-19?” on a scale from 1 (extremely unlikely) to 5 (extremely likely)”. In compliance with the policy of the Journal of Risk and Uncertainty ordinal scores were converted as follows: 1 (extremely unlikely) and 2 (unlikely) were converted to 0; 3 (neither likely nor unlikely) was converted to 1, and 4 (likely) and 5 (extremely likely) were converted to 2.Five hundred of our participants were additionally asked the original question regarding danger to the health of the human population. In compliance with the policy of the Journal of Risk and Uncertainty ordinal scores were converted as follows: scores below the midpoint of the scale (< 50) were converted to 0 and those above (> = 50) were converted to 1.
2. We added a question to assess “perceived absolute personal risk” by asking participants “How likely do you think you are to get COVID-19?” on a scale from 1 (extremely unlikely) to 5 (extremely likely)”. In compliance with the policy of the Journal of Risk and Uncertainty ordinal scores were converted as follows: 1 (extremely unlikely) and 2 (unlikely) were converted to 0; 3 (neither likely nor unlikely) was converted to 1, and 4 (likely) and 5 (extremely likely) were converted to 2. Many of the items introduced in the survey completed by subjects in time 1 for the additional parallel studies not reported here were not introduced in time 2.

### Analysis, time 1

Three separate binomial logistic regressions were run to assess the effect of perceived relative personal risk and perceived public risk on happiness, anxiety and behavioral compliance controlling for all demographics (age, gender, ethnicity, household income, healthcare insurance satisfaction, political orientation, level of education, children, state restrictions). The same logistic regression models were run again with sense of control as an additional predictor. We also performed two separate orded logit regression models to predict perceived relative personal risk from sense of control controlling for all demographics, and two separate binominal logistic regression models to predict perceived public risk from all demographics and from sense of control controlling for all demographics.

Mediation analyses were performed (Hayes, 2009) to assess the relationship between sense of control, perceived relative personal risk, perceived public risk and the dependent variables. We performed a Sobel test to determine whether the addition of the indirect pathway significantly reduced the direct pathway.

### Analysis, time 2

Analysis was the same as in time 1 except for the following changes: We assessed perceived public risk using participants’ rating of “How likely do you think a person is to get COVID-19?”. Logistic regression analyses were performed controlling for all demographics except state restrictions, as level of restrictions after converting to 0 and 1 was constant across all states at the time of data collection (1 for all states). All analyses were repeated, replacing perceived relative personal risk with perceived absolute personal risk to examine if results differ. Finally, to examine if any of the main variables altered during the month of lockdown, temporal effects were inspected. Related-Samples McNemar’s Change Tests were computed to assess the temporal change between timepoint 1 and timepoint 2 for anxiety, happiness, compliance, sense of control, and perceived public risk and a Friedman’s test to assess the temporal change for perceived relative personal risk (as this variable had 3 possible values rather than 2).

## Results, time 1

### Perceived relative personal risk is low and perceived public risk is high

**Fig. 2:**
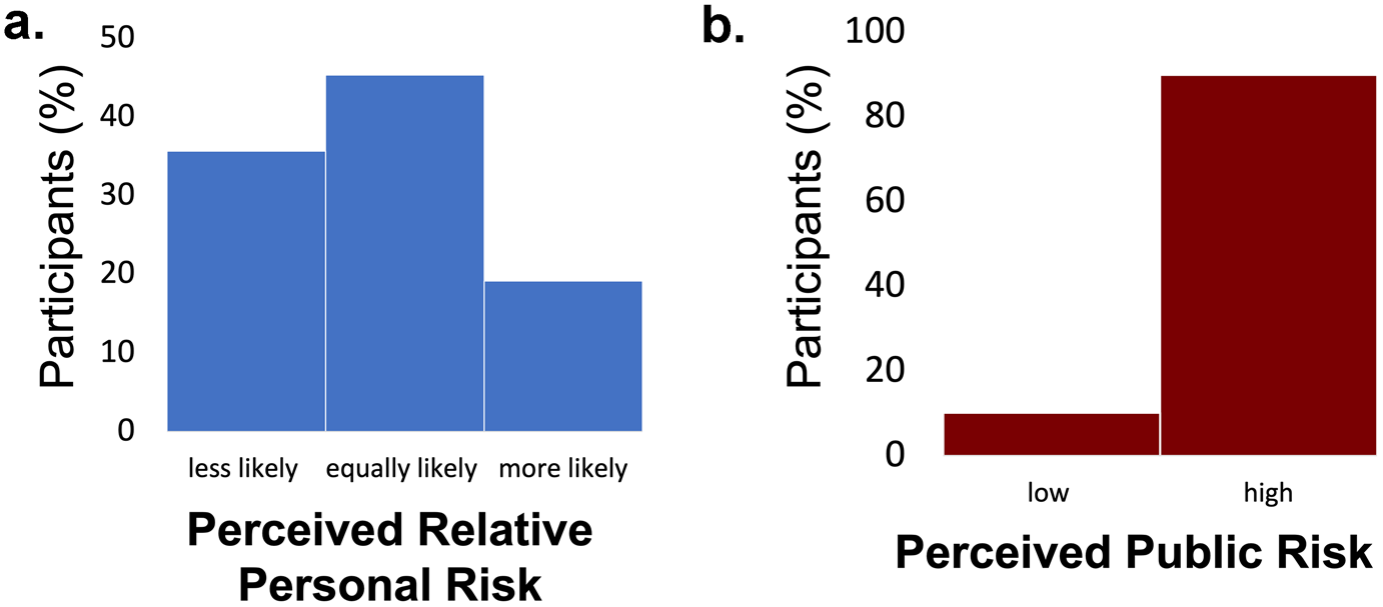
**Perceived relative personal risk is low and perceived public risk is high. **(**a**) Displayed are percentages of participants who perceived themselves to be less likely than others their age and gender to be infected by COVID, as likely, or more likely (blue). (**b**) Displayed are percentages of participants who perceived COVID to pose a low threat to the health of the population and high threat (red)

People’s estimate of relative personal risk was positively skewed, while their perception of public risk was negatively skewed. In particular, 35.6% of participants indicated they believed they were less likely to get COVID-19 relative to others of their same age and gender, while only 19.1% indicated they believed they were more likely to get COVID-19 relative to others their age and gender. Almost half (45.3%) indicated they believed they were as likely to get COVID-19 as others their age and gender (Fig. 2a). As for public risk, the vast majority (89.9%) indicated they believed COVID-19 posed high danger to the health of the human population (Fig. 2b).

**Fig. 3:**
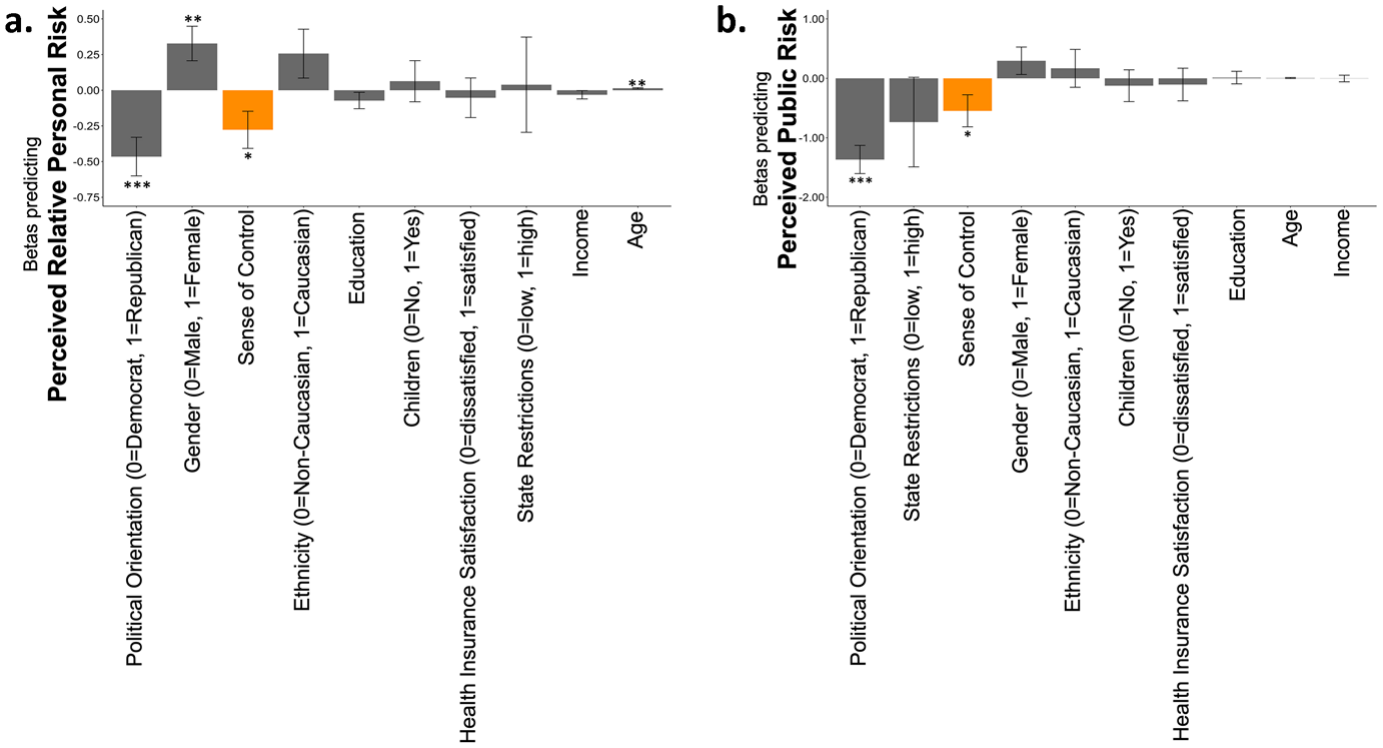
**High sense of control is associated with low risk perception** (time 1). Displayed are the Beta coefficients from logistic regressions predicting (**a**) perceived relative personal risk (that is “Relative to others of your age and gender do you think you are less/more likely to get COVID-19?”and (**b**) perceived public risk (that is “Do you think COVID-19 presents a real danger to the health of the human population?”). (**a**) People with strong sense of control (orange bar) were more likely to perceive relative personal risk as low, as were males, younger individuals, and Republicans. (**b**) People with strong sense of control (orange bar) were more likely to perceive public risk as low, as were Republicans. Regressors are ordered from the largest magnitude to the smallest. *p < 0.05, **p < 0.001, *** *p* < 0.0001, Error Bars SEM

We next examined which factors were related to participants’ risk estimations. To that end we ran two logistic regressions – one predicting perceived relative personal risk and one predicting perceived public risk. The independent factors included were demographics (age, gender, income, education, ethnicity, number of children), political orientation, restriction in the participant’s state, satisfaction with health insurance as well as sense of control measured using the Sense of Control Scale (Lachman & Weaver, 1998). This questionnaire examines people’s sense of mastery and perceived constraints. Interestingly, only two factors predicted both perception of personal and public risk: political orientation (perceived relative private risk: β = -0.465, p < 0.001, perceived public risk β = -1.366, p < 0.001, also see Bruine de Bruin et al., 2020 for the importance of political orientation on COVID-19 risk estimates) and a sense of control (perceived relative private risk: β = -0.28, p = 0.033, Fig. 3a, Table S1**;** perceived public risk: β = -0.546, p = 0.043, Fig. 3b, Table S2). In particular, both Republicans and those who scored high on the sense of control questionnaire tended to perceive personal, and public risk as low. In addition, males (β = 0.328, p = 0.007) and younger individuals (β = 0.014, p = 0.001) were all more likely to perceive their relative personal risk as low. No other factor was significantly associated with perceived public risk. Note, that all factors are included in one regression, thus the influence of one factor is controlled for when examining the other.

### Perceived relative personal risk, but not perceived public risk, is related to participants’ happiness

**Fig. 4:**
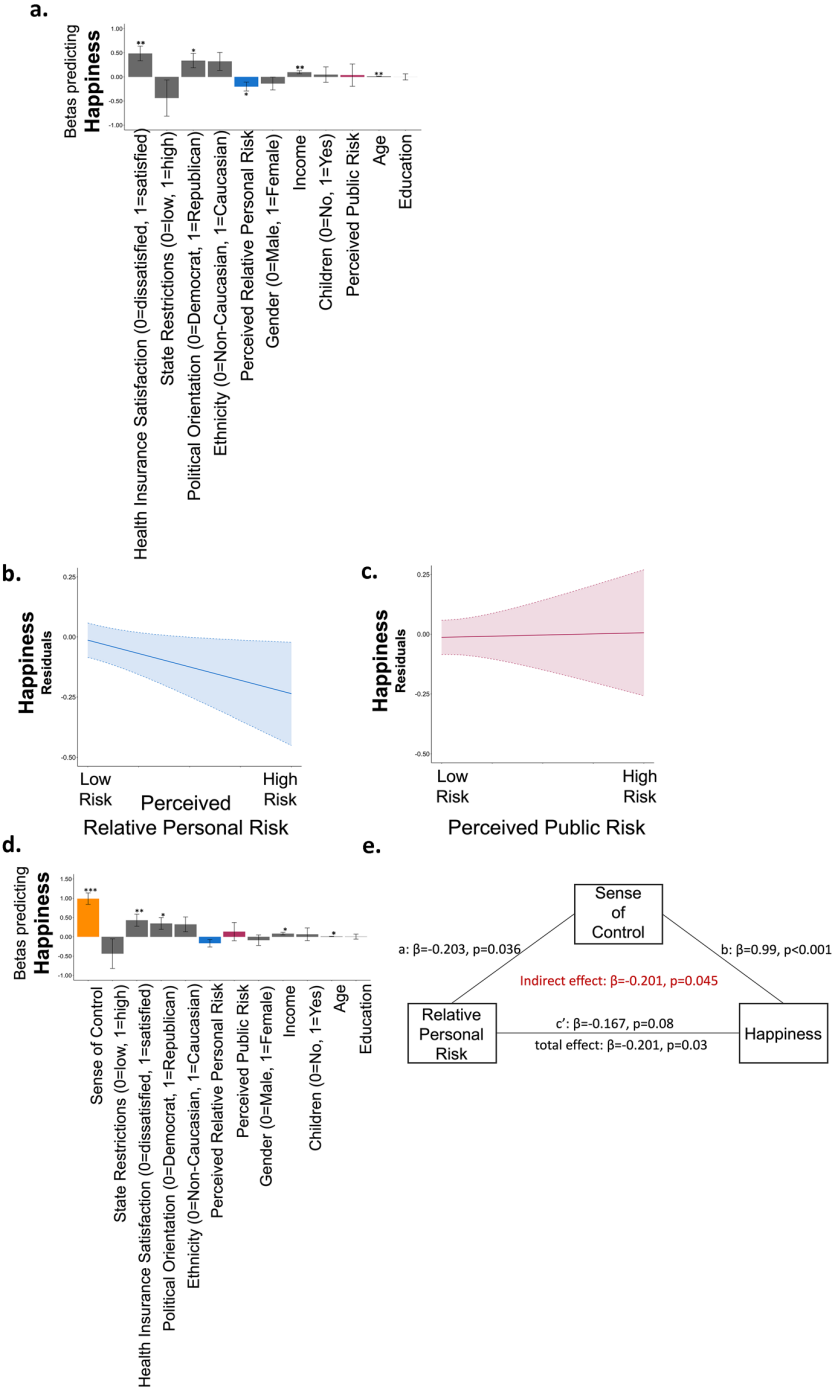
**Perception of relative personal risk is related to happiness (time 1). **(**a**) Displayed are the Beta coefficients from a logistic regression predicting happiness, which shows that those who report low perceived relative personal risk (blue) are happier. Perceived public risk (red) is not associated with happiness. These associations are also portrayed in (**b **&** c**). Here, the Y and X axis display residuals from the same model, which includes all demographic controls. Clouds represent confidence intervals. (**d**) Adding sense of control (orange) to the model reveals that sense of control is the strongest factor predicting happiness and reduces the association between perceived relative personal risk and happiness. Indeed, a formal mediation analysis shows that (**e**) sense of control mediated the relationship between perceived relative personal risk and happiness. Regressors are ordered from the largest magnitude to the smallest. *p < 0.05, **p < 0.001, ***p < 0.0001, Error Bars SEM

We were interested whether and how perceived risk was related to peoples’ sense of happiness in times of crisis. We thus asked our participants to indicate “how happy are you with your life these days?” on a scale from least happy time of my life to most happy time of my life. About half (52%) of the participants rated their happiness as lower than other times in their life. Importantly, people who perceived their relative personal risk as lower than others their age and gender were more likely to indicate they were happier (Beta from a logistic regression model predicting relative happiness from perceived relative personal risk, perceived public risk and all demographic variables as controls β = -0.201, p = 0.03, Fig. 4a, b, Table S3). Perceived public risk on the other hand was not associated with happiness (Beta from the same model β = 0.036, p = 0.877, Fig. 4a, c). While the result is correlational, it is plausible that the belief that one is relatively immune to COVID-19 protected people’s sense of happiness during the crisis and/or that happiness alters personal risk perception.

Adding “sense of control” into the previous regression revealed that sense of control was the variable most strongly associated with happiness. The Beta coefficient signifying the magnitude of the relationship between sense of control and happiness (β = 0.99, p < 0.001) was more than double that of any other variable. In addition to a high sense of control those who were satisfied with health insurance (β = 0.43, p = 0.006), older individuals (β = 0.011, p = 0.025), those with higher income (β = 0.084, p = 0.011) and Republicans (β = 0.347, p = 0.023), all reported greater happiness (Fig. 4d, Table S4). We note that all these factors were included in one model, thus the results indicate independent effects of each variable.

It has been shown that sense of control is related to both optimism and happiness (Lachman & Weaver, 1998; Larson, 1989; Seligman, 2011). We thus examined if the relationship between perceived relative personal risk and happiness was mediated by participants’ sense of control. To that end, we computed a mediation model, controlling for all demographic variables (see Sect. 1). The model first confirmed that perceived relative personal risk was negatively related to happiness (total effect: β = -0.201, p = 0.03). Importantly, this relationship was mediated by the sense of control (indirect effect: β = -0.201, *p* = 0.045, *Sobel Test: z* = -2.009). Indeed, once the sense of control was statistically accounted for, the relationship between perceived relative personal risk and happiness was reduced to trend level (c’: β = -0.167, *p* = 0.08). In contrast, sense of control predicted happiness even when perceived relative personal risk was accounted for (path b: β = 0.99, *p* < 0.001). The reverse mediation was not significant. That is perceived relative personal risk did not mediate the relationship between sense of control and happiness (total effect: β = -0.90, *p* < 0.001; indirect effect: β = -0.034 *p* = 0.24, Sobel Test: z = 1.176; path c’: β = 0.99, p < 0.001; path a: β = -0.254, *p* = 0.051; path b: β = -0.167, *p* = 0.08). These findings suggest that a sense of control mediates its relationship with happiness, but not vice versa.

### Perceived relative personal risk and perceived public risk are related to anxiety

**Fig. 5:**
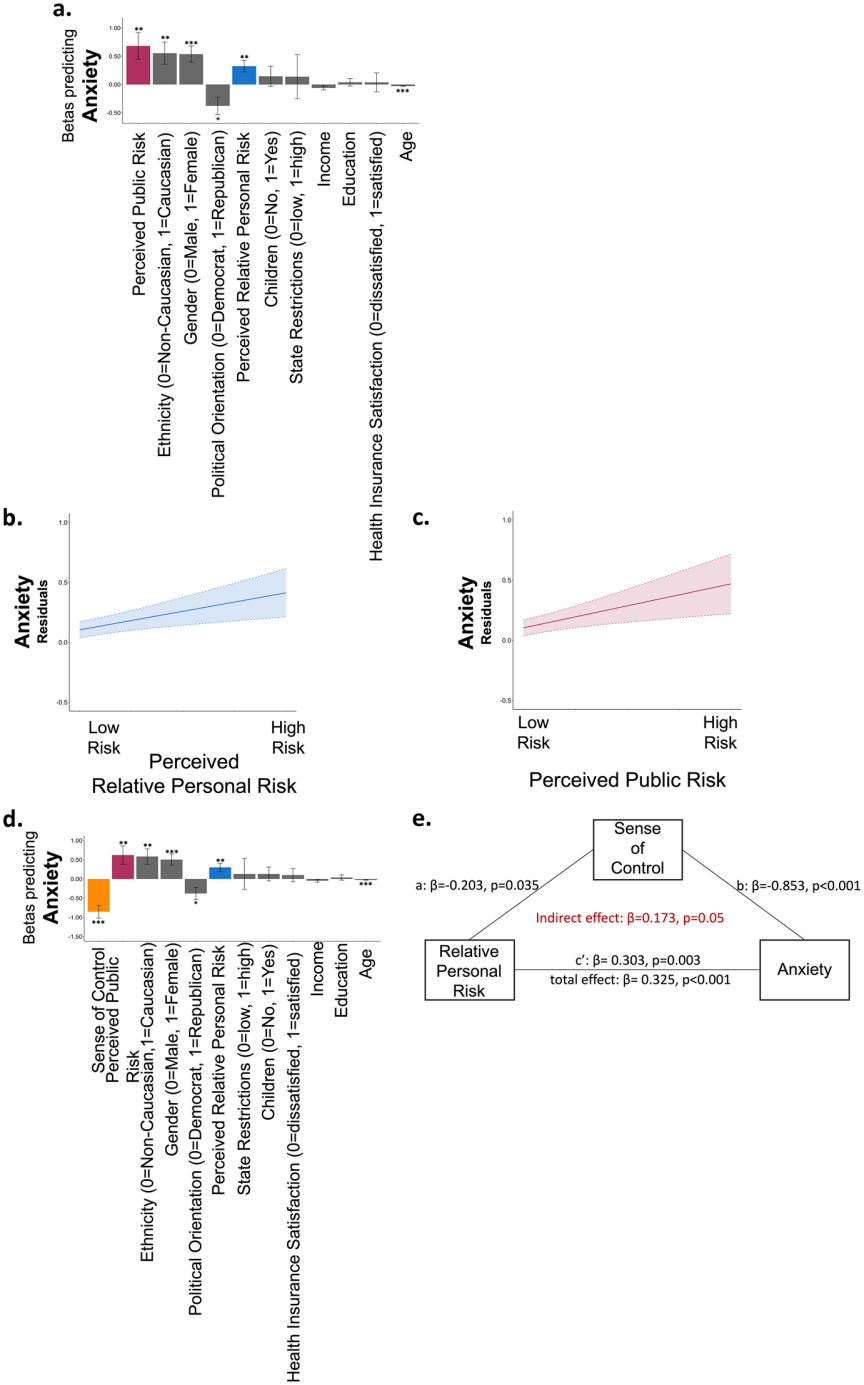
**Perception of risk to self and others is associated with anxiety (time 1). **(**a**) Displayed are the Beta coefficients from a logistic regression predicting anxiety (STAI scores), which shows that those who report higher perceived relative personal risk (blue) and perceived public risk (red) reported greater anxiety. These associations are also portrayed in (**b **&** c**). Here, the Y and X axis display residuals from the same model, which includes all demographic controls. Clouds represent confidence intervals. (**d**) Adding sense of control (orange) to the model reveals that sense of control is the strongest factor predicting anxiety and reduces the association between perceived relative personal risk and anxiety. Indeed, a formal mediation model shows that (**e**) sense of control partially mediated the relationship between perceived relative personal risk and anxiety. Regressors are ordered from largest magnitude to smallest magnitude. *p < 0.05, **p < 0.001, ***p < 0.0001, Error Bars SEM

Thus far we have shown that perceived relative personal risk, but not perceived public risk, was associated with happiness during the COVID-19 crisis. We next examined if these factors were related to anxiety, which we measured using the short version of STAI (Marteau & Bekker, 1992). We found that perceived risk, both personal and public, were strongly associated with high anxiety (Betas from a logistic regression including all demographics, perceived relative personal risk: β = 0.325, *p* = 0.001, Fig. 5a, b, perceived public risk: β = 0.681, *p* = 0.004, Fig. 5a, c, Table S5). This was true also when adding sense of control into the model, which in itself was negatively associated with anxiety and was the strongest predictor of it (β = -0.854, *p* < 0.001). In addition, younger individuals, females, Caucasians, and Democrats were more anxious (age: β = -0.027, *p* < 0.001; gender: β = 0.505, *p* < 0.001, ethnicity: β = 0.582, *p* = 0.004, political orientation: β = -0.379, *p* = 0.016, Fig. 5d, Table S6).

To examine if the relationship between perceived relative personal risk and anxiety was mediated by participants’ sense of control, we computed a mediation model, controlling for all demographic variables (see Sect. 1). Once again, perceived relative personal risk was positively related to anxiety (total effect: β = 0.325, p < 0.001). This relationship was partially, but not fully, mediated by a sense of control (indirect effect: β = 0.173, *p* = 0.05, *Sobel Test: z* = 1.9470), as after accounting for a sense of control perceived relative personal risk was still related to anxiety (c’: β = 0.303, *p* = 0.003). By contrast, we did not find evidence for the reverse mediation. That is perceived relative personal risk did not mediate the relationship between sense of control and anxiety (total effect: β = -0.876, *p* < 0.001; path c’: β = -0.854, p < 0.001; indirect effect: 0.062, *p* = 0.163, *Sobel Test: z* = 1.395, path a: β = -0.254, *p* = 0.051; path b: β = 0.302, *p* = 0.003). Sense of control did not mediate the relationship between perceived public risk and anxiety, although a trend was observed (indirect effect: 0.426, *p* = 0.083, *Sobel Test: z* = 1.732) which became significant in Time 2 (see Online Appendix).

### Perceived public risk, but not perceived relative personal risk, is associated with behavioral compliance

The above results show that perceived risk is associate with people’s emotional state during the pandemic. We next examined whether it is also associated with people’s behavioral response to it. To that end, we assessed participants’ self-reported behavioral compliance with government officials’ advice to mitigate the COVID-19 outbreak. We found that behavioral compliance was high: 97% of participants reported putting effort into social distancing, 95% reported putting effort into frequent hand washing and 77% into avoiding face touching, 95% reported they did not visit other people’s homes in the last week and 82% reported they have not come within 1 m of people outside their own residence.

**Fig. 6:**
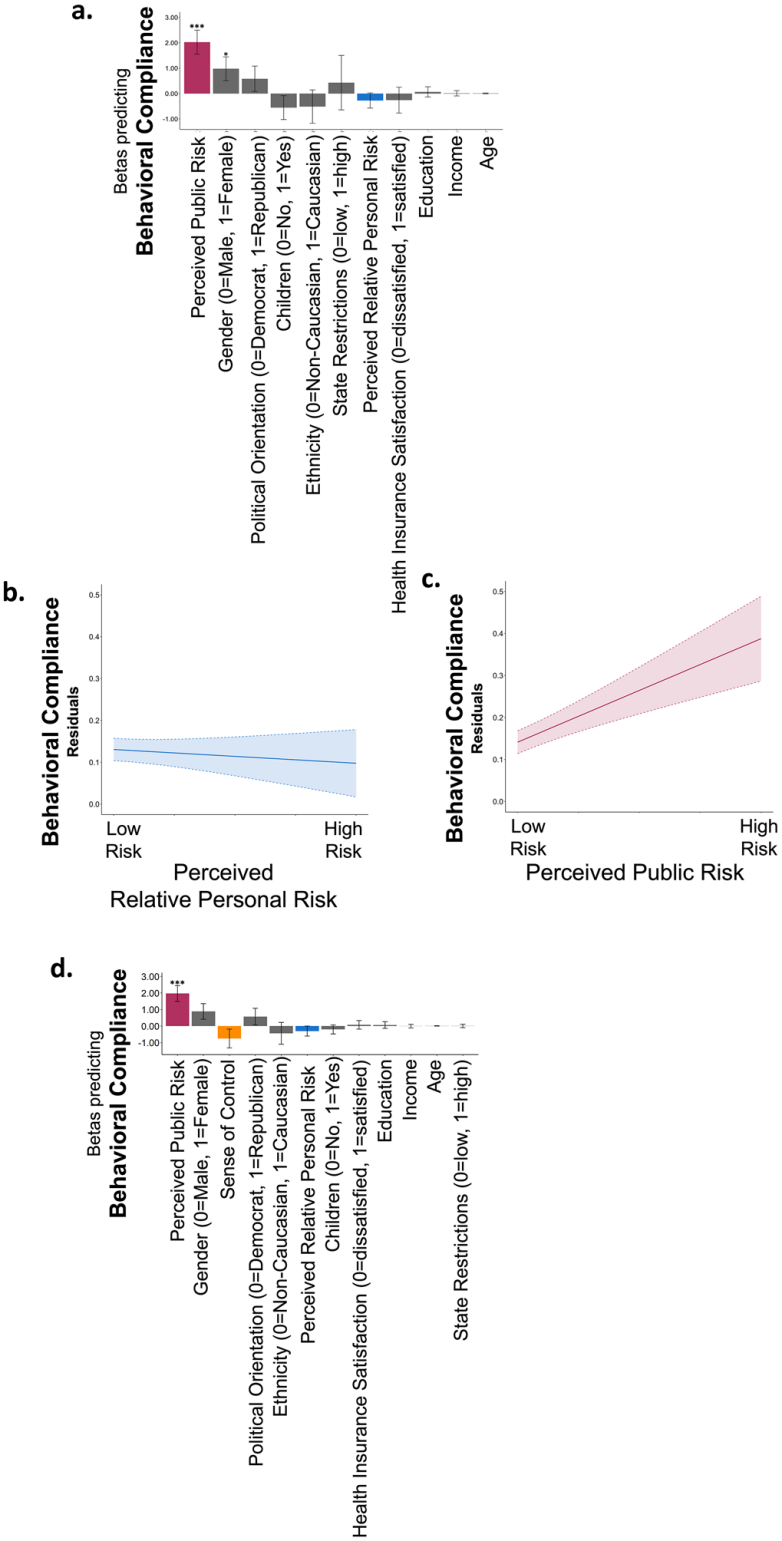
**Behavioral compliance is associated with perception of public risk, but not relative personal risk (time 1). **(**a**) Displayed are the Beta coefficients from a logistic regression predicting behavioral compliance, which shows that those who report higher perceived public risk (red) are more likely to comply. Perceived relative personal risk (blue), however, is not associated with behavioral compliance. These associations are also portrayed in (**b **&** c**). Here, the Y and X axis display residuals from the same model, which includes all demographic controls. Clouds represent confidence intervals. (**d**) Adding sense of control (orange) to the model reveals that sense of control is not related to behavioral compliance. Regressors are ordered from largest magnitude to smallest magnitude. *p < 0.05, ***p < 0.0001, Error Bars SEM

We averaged participants’ raw scores on all these measures such that each participant had one score reflecting behavioral compliance. Following the guidelines of the Journal of Risk and uncertainty this variable was binarized with 0 summarizing responses below the midpoint of the scale (< 50) and 1 summarizing responses equal or above midpoint of the scale. These scores were then entered as a dependent variable in a binomial logistic regression model. We found that while perceived public risk was strongly related with behavioral compliance (Beta from a logistic regression model including perceived relative personal risk, perceived public risk and all demographic controls: β = 2.03, *p* < 0.001, Fig. 6a, c, Table S7), perceived relative personal risk was not (β = -0.272, *p* = 0.362, Fig. 6a, b). Adding sense of control into the model did not alter the results (β = 1.946, *p* < 0.001), which in itself was not related to behavioral compliance (β = -0.725, *p* = 0.203, Fig. 6d, Table S8). The relationship between perceived public risk and behavioral compliance could not be explained by high anxiety alone, as even when we add anxiety into the model, the effect of perceived public risk on behavioral compliance remains significant (Betas in a model including perceived relative personal risk, perceived public risk, anxiety and all demographic controls Anxiety: β = 0.429, p = 0.34, Perceived public risk: β = 1.98, p < 0.001, Table S9).

## Results, time 2

In time 1 we found divergent effects of perceived relative personal risk and perceived public risk on happiness and behavioral compliance and converging effects on anxiety. To assess perceived relative personal risk in time 1 we asked participants about their risk of getting infected relative to others. To assess perceived public risk, however, we asked about whether they believed the virus presented a danger to the health of the human population. It is possible that assessing perceptions of “infection risk” vs. “danger” was driving the results rather than assessing perception related to the self vs. the human population. Thus in time 2 (N = 683) we assessed perceived relative personal risk as before but to assess perceived public risk we now asked “How likely do you think *a person* is to get COVID-19?” on a scale from 1 (extremely unlikely) to 5 (extremely likely)”. We also assessed “perceived absolute personal risk” by asking participants “How likely do you think you are to get COVID-19?” on a scale from 1 (extremely unlikely) to 5 (extremely likely). These additional questions enabled us to assess perceived personal and public risk using similar wording of the questions. Following guidelines of the Journal of Risk and Uncertainty, both variables were recoded with 1 and 2 (extremely unlikely/unlikely to get COVID-19) coded as 0; 3 (neither unlikely nor likely to get COVID-19) coded as 1; and 4 and 5 (likely/extremely likely to get COVID-19) coded as 2.

The subjects we tested in time 2 were 683 of the same subjects we ran in time 1, tested one month later. Moreover, we were able to examine for changes over the month of lockdown. As we report below key results observed at time 1 are replicated in Time 2.

## Results of time 2 replicate those observed at time 1

### Perceived relative personal risk is low and perceived public risk is high

People’s estimate of relative personal risk was positively skewed, while their perception of public risk was negatively skewed. In particular, 34.6% of participants indicated they believed they were less likely to get COVID-19 relative to others of their same age and gender, while only 18.6% indicated they believed they were more likely to get COVID-19 relative to others their age and gender. Almost half (46.8%) indicated they believed they were as likely to get COVID-19 as others their age and gender. With regards to public risk, the majority of participants (60.8%) indicated a person’s likelihood of getting COVID-19 was extremely likely/likely, with only 18.7% indicating they thought it was extremely unlikely/unlikely and 20.5% indicating it was neither likely nor unlikely. In contrast, only 31.2% considered their likelihood of getting COVID-19 as extremely/ high, while 42.3% of participants considered their likelihood of getting COVID-19 as extremely/very low and 26.5% considered themselves neither likely nor unlikely to get COVID-19 and. Sense of control was associated with both perceived relative personal risk (β = -0.535, *p* = 0.001, Online Appendix Fig. 1a, Table S12) and perceived public risk (β = -0.59, *p* = 0. 0021 Online Appendix Fig. 1b, Table S13) and trend level with perceived absolute personal risk (β = -0.285, *p* = 0.078, Table S14). For full details and statistics of time 2 see Online Appendix.

Perceived relative personal risk was associated with happiness (perceived relative personal risk: β = -0.298, *p* = 0.011, Online Appendix Fig. 2a, b, Table S16) while perceived public risk was not (β = -0.145, *p* = 0.179, Online Appendix Fig. 2a, c). Anxiety was associated with both perceived relative personal risk (β = 0.384, *p* = 0.002, Online Appendix Fig. 3a, b, Table S19) and perceived public risk: β = 0.249, *p* = 0.022, Online Appendix Fig. 3a, c). Absolute risk was associated with anxiety at a trend level (β = 0.204, *p* = 0.063, Table S21) and not associated with happiness (β = -0.149, *p* = 0.158, Table S18). This finding is in accord with past suggestions that comparison to others can be more important for a person’s affective state than absolute measures (e.g., Boyce et al., 2010). Behavioral compliance was associated with perceived public risk (β = 1.023, *p* = 0.01, Online Appendix Fig. 5a, c, Table S22), but not perceived relative personal risk (β = -0.084, *p* = 0.854, Online Appendix Fig. 5a, b) or absolute risk (β = -0.519, *p* = 0.198, Table S24).

### Over the month of lockdown well-being increased

**Fig. 7:**
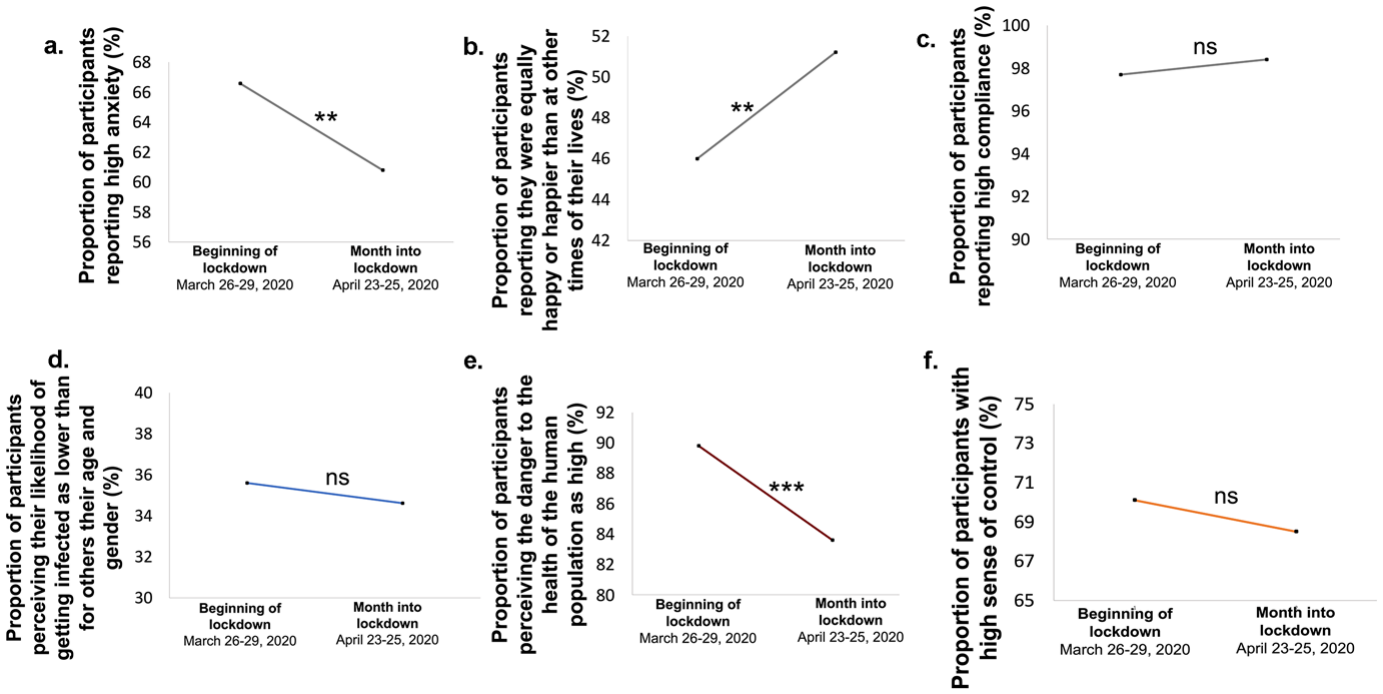
**People adapt to lockdown**. We tested participants at two times; around the time lockdown was imposed (time 1, March 26–29, 2020) and one month into lockdown (time 2, April 23–25, 2020). We found that (**a**) the proportion of participants reporting high anxiety decreased one month into lockdown. (**b**) The proportion of participants reporting they were as or more happy than other times of their lives increased. (**c**) Behavioral Compliance (**d**) perceived relative personal risk and (**f**) sense of control all remained stable. (**e**) The proportion of participants perceiving the danger of COVID-19 to humanity to be high decreased

Examining subjects’ responses at the beginning of lockdown and again a month into lockdown revealed a positive change in well-being. First, a month in, a smaller proportion of participants reported high anxiety (60.8%) compared to when states were just entering lockdown (66.6%, X^2^(1, n = 683) = 12.071, p = 0.001, Fig. 7a). Moreover, a greater proportion of participants reported they were happier than at other times of their lives (51.2%) compared to the beginning of the crisis (46%, X^2^(1, n = 683) = 6.731, p = 0.009, Fig. 7b). These results align with past studies showing that humans adapt well to adversities and environmental change (Bonanno et al., 2002, 2004; Brickman et al., 1978; Dijkers, 1997; Frederick & Loewenstein, 1999). Additionally, a smaller proportion of participants perceived the danger of COVID-19 to humanity to be high (83.6%) compared to the beginning of lockdown (89.8%, X^2^(1, n = 500) = 15.754, p < 0.001, Fig. 7e). There was no change in the proportion of participants who reported a high sense of control (Time 1: 70.1%, time 2: 68.5%, X^2^(1, n = 683) = 0.513, p = 0.474, Fig. 7f), no change in compliance (high compliance at time 1 = 97.7%; and time 2 = 98.4%, X^2^(1, n = 683) = 1.231, p = 0.267, Fig. 7c), nor in how people perceived their relative personal risk (X^2^(2, n = 683) = 0.017, p = 0.846, Fig. 7d).

## Discussion

Surveying a representative sample of Americans over two time points during the pandemic of 2020 we found that many people believed COVID-19 posed a significant health danger to humans and perceived the risk of an average person to get COVID-19 as high. However, the majority did not consider their own risk to be high in absolute terms, nor higher relative to others their age and gender (see also Kuper-Smith et al., 2020; Wise et al., 2020). The question we posed was how these different perceptions of risk were related to psychological well-being and behavior. We found that individuals who believed they were less at risk of being infected by COVID-19 than others were happier. However, believing COVID-19 posed a great danger to humanity was unrelated to people’s happiness. This suggest that one’s perceived risk relative to others is especially important for people’s positive affect, while perceived risk to humanity is not. At the same time, both risk perceptions were related to anxiety. People who believed COVID-19 was a risk to themselves and/or to the human race tended to be more anxious. This suggests that while perceived relative personal risk relates to both positive and negative affect, perceived public risk relates only to negative affect.

Surprisingly, the likelihood that individuals complied with behavioral recommendations to mitigate the risk of COVID-19 was related to their views regarding the danger the virus poses to people in general, but not significantly related to whether they believed they themselves were at high risk. That is, people who believed the virus posed a great danger to humanity reported putting greater effort in social distancing, handwashing and avoiding touching their faces. Believing that the virus posed an especially high risk to the self was, however, unrelated to these behaviors. This effect remained even when accounting for anxiety and replicated across both time points. We thus speculate that the main motive for behavioral compliance was reducing the risk to the population as a whole, rather than the risk to the self. This in accordance with recent findings suggesting that public health messaging which focus on the concern for the greater good and responsibilities towards others may help induce behavioral compliance (Everett et al., 2020). Indeed, this may explain the success of the British Government’s message during the pandemic—"Stay Home. Protect the NHS. Save Lives” -which focused not on saving oneself but on saving “lives” and saving the national health service.

We tested the same subjects across two times—at the beginning of the crisis when states were moving into lockdown (time 1) and a month later when states were under lockdown (time 2). Interestingly, we observed a positive change in subjects’ well-being. A month into lockdown anxiety was lower than it was when states were just entering lockdown, happiness was greater and participants’ perception of the danger of COVID-19 to humanity was lower. These results align with past studies showing that humans adapt well to adversities and environmental change (Bonanno et al., 2002, 2004; Brickman et al., 1978; Dijkers, 1997; Frederick & Loewenstein, 1999).

In general people exhibited significant resilience during the pandmic with half of the population indicating they were as, or more happy as other times in their lives. The strongest predictor of happiness was a sense of control. Those who believed they had agency over their own life were happier, less anxious and perceived the danger COVID poses to themsleves and others as lower. In fact, the positive relationship between perceived personal risk and happiness was partially related to people’s sense of control. These results align with the suggestions that a sense of control is related to both optimism (Harris, 1996; Helweg-Larsen & Shepperd, 2001; Klein & Helweg-Larsen, 2002; Zakay, 1984) and overall well-being (Lachman & Weaver, 1998; Larson, 1989).

Here we examined people’s attitudes regarding a specific threat—COVID-19. There is some evidence, however, that the results generalize to other threats. For example, following the financial collapse of 2008 polls showed that people were pessimistic about the financial future of their country, less so about their own financial prospects (Ipsos MORI, 2008). Moreover, people perceive their fatality risk from natural disasters as below average (Viscusi & Zeckhauser, 2006), and with regards to climate change, people express little concern about the likely effects of climate change in their own region, but are more pessimistic with regards to the effects on their nation and the planet as a whole (Dunlap and Gallup, 1993). Such markedly different estimates of risk to oneself and others may arise because when estimating their own risk individuals assign more weight to their direct experience than to the experience of others (Viscusi & Zeckhauser, 2015). In other words, as most individuals were not infected by COVID themselves nor experienced a natural disaster, they estimate their own future risks in these domains as lower than the known risk in the population. Another factor contributing to this dissociation is people’s tendency to be overly optimistic about their own prospects relative to others (Sharot, 2011; Weinstein, 1980), which arises as people update their beliefs about their own vulnerability to a larger extent when receiving good news than when receiving bad news (Sharot et al., 2011). Moreover, past studies have shown that different moderators influence people’s perception of risk to oneself and to others, with factors associated with negative affect and sense of control primarily influencing personal risk estimates (for review see Helweg-Larsen & Shepperd, 2001).

Future studies are needed to examine whether the effects on well-being and behavior reported in this study generalize to other threats such as war, financial collapse and climate change. The results of such studies would be important for predicting the impact of such threats on people’s well-being and for understanding when and why people are likely to change their behavior to mitigate risk. For example, similar to the findings reported here, it is possible that the likelihood that people make “green choices” is related to their belief that climate change poses a threat to humanity, regardless of whether they believe it poses a perceived relative personal risk. Such knowledge can be useful for advocates and policy makers in framing information to nudge individuals to select actions that protect themselves and others from natural and man-made threats.

## Supplementary information

Below is the link to the electronic supplementary material.

Supplementary file1 (PDF 1868 KB)

## Data Availability

All files will be made available on Github https://github.com/affective-brain-lab/Globig_PersonalPublicRisk upon publication.

## References

[CR1] Bonanno GA, Wortman CB, Lehman DR, Tweed RG, Haring M, Sonnega J, Nesse RM (2002). Resilience to loss and chronic grief: A prospective study from preloss to 18-months postloss. Journal of Personality and Social Psychology.

[CR2] Bonanno GA, Wortman CB, Nesse RM (2004). Prospective patterns of resilience and maladjustment during widowhood. Psychology and Aging.

[CR3] Boyce CJ, Brown GD, Moore SC (2010). Money and happiness: Rank of income, not income, affects life satisfaction. Psychological Science.

[CR4] Brickman P, Coates D, Janoff-Bulman R (1978). Lottery winners and accident victims: Is happiness relative?. Journal of Personality and Social Psychology.

[CR5] Bruine de Bruin W, Saw HW, Goldman DP (2020). Political polarization in US residents’ COVID-19 risk perceptions, policy preferences, and protective behaviors. Journal of Risk and Uncertainty.

[CR6] Dember WN, Penwell L (1980). Happiness, depression, and the Pollyanna principle. Bulletin of the Psychonomic Society.

[CR7] Dijkers M (1997). Quality of life after spinal cord injury: A meta analysis of the effects of disablement components. Spinal Cord.

[CR8] Dunlap, R. E., Jr., G. H. G., & Gallup, A. M. (1993). Of global concern. *Environment: Science and Policy for Sustainable Development*, *35*(9), 7–39. 10.1080/00139157.1993.9929122

[CR9] Everett, J. A., Colombatto, C., Chituc, V., Brady, W. J., & Crockett, M. J. (2020). The effectiveness of moral messages on public health behavioral intentions during the COVID-19 pandemic. *PsyArXiv*, 1–23. 10.31234/osf.io/9yqs8

[CR10] Frederick, S., & Loewenstein, G. (1999). 16 Hedonic adaptation. *Well-Being: The Foundations of Hedonic Psychology*, 302–329.

[CR11] Hale, T., Atav, T., Hallas, L., Kira, B., Phillips, T., Petherick, A., & Pott, A. (2020). Variation in US states responses to COVID-19. *Blavatnik School of Government*.

[CR12] Harris P (1996). Sufficient grounds for optimism? The relationship between perceived controllability and optimistic bias. Journal of Social and Clinical Psychology.

[CR13] Hayes AF (2009). Beyond Baron and Kenny: Statistical mediation analysis in the new millennium. Communication Monographs.

[CR14] Helweg-Larsen M, Shepperd JA (2001). Do moderators of the optimistic bias affect personal or target risk estimates? A review of the literature. Personality and Social Psychology Review.

[CR15] Ipsos MORI. (2008). Political Monitor, March 2008, [UK]. . Retrieved from: https://www.ipsos.com/ipsos-mori/en-uk/ipsos-mori-political-monitor-march-2008

[CR16] Klein CTF, Helweg-Larsen M (2002). Perceived control and the optimistic bias: A meta-analytic review. Psychology and Health.

[CR17] Krieger JL, Murray F, Roberts JS, Green RC (2016). The impact of personal genomics on risk perceptions and medical decision-making. Nature Biotechnology.

[CR18] Kuper-Smith, B. J., Doppelhofer, L., Oganian, Y., Rosenblau, G., & Korn, C. W. (2020). Optimistic beliefs about the personal impact of COVID-19. *PsyArXiv*, 1–4. Retrieved from https://psyarxiv.com/epcyb/

[CR19] Kuzmanovic B, Jefferson A, Vogeley K (2015). Self-specific optimism bias in belief updating is associated with high trait optimism. Journal of Behavioral Decision Making.

[CR20] Lachman ME, Weaver SL (1998). The sense of control as a moderator of social class differences in health and well-being. Journal of Personality and Social Psychology.

[CR21] Langer EJ (1975). The illusion of control. Journal of Personality and Social Psychology.

[CR22] Larson R (1989). Is feeling “in control” related to happiness in daily life?. Psychological Reports.

[CR23] Marteau TM, Bekker H (1992). The development of a six-item short-form of the state scale of the Spielberger State—Trait Anxiety Inventory (STAI). British Journal of Clinical Psychology.

[CR24] Seligman, Martin E P. (2011). *Learned Optimism: How to Change Your Mind and Your Life*. Vintage.

[CR25] Sharot T, Korn CW, Dolan RJ (2011). How unrealistic optimism is maintained in the face of reality. Nature Neuroscience.

[CR26] Sharot T (2011). The optimism bias. Current Biology.

[CR27] Spielberger, C. D. (2010). State‐Trait anxiety inventory. *The Corsini Encyclopedia of Psychology*, 1.

[CR28] Strunk, D. R., Lopez, H., & DeRubeis, R. J. (2006). Depressive symptoms are associated with unrealistic negative predictions of future life events. *Behaviour Research and Therapy*, *44*(6), 861–882. 10.1016/j.brat.2005.07.001 10.1016/j.brat.2005.07.001 16126162

[CR29] Tobias-Webb J, Limbrick-Oldfield EH, Gillan CM, Moore JW, Aitken MRF, Clark L (2017). Let me take the wheel: Illusory control and sense of agency. Quarterly Journal of Experimental Psychology.

[CR30] Viscusi WK, Zeckhauser RJ (2006). National survey evidence on disasters and relief: Risk beliefs, self-interest, and compassion. Journal of Risk and Uncertainty.

[CR31] Viscusi WK, Zeckhauser RJ (2015). The relative weights of direct and indirect experiences in the formation of environmental risk beliefs. Risk Analysis.

[CR32] Weinstein ND (1980). Unrealistic optimism about future life events. Journal of Personality and Social Psychology.

[CR33] Wise, T., Zbozinek, T. D., Michelini, G., Hagan, C. C., & Mobbs, D. (2020). Changes in risk perception and protective behavior during the first week of the COVID-19 pandemic in the United States. *PsyArXiv [Working Paper]*, (4), 1–13. 10.31234/OSF.IO/DZ428 10.1098/rsos.200742 PMC7540790 33047037

[CR34] Zakay D (1984). The influence of perceived event’s controllability on its subjective occurrence probability. The Psychological Record.

