# Supplementary material for: Perceptions of personal and public risk: Dissociable effects on behavior and well-being
Source: J Risk Uncertain. 2022 Apr 2;64(2):213–34. doi: 10.1007/s11166-022-09373-0 (PMC8976215; doi:10.1007/s11166-022-09373-0)
Supplement: Supplementary file 1 — Supplementary file1 (PDF 1868 KB) [file 11166_2022_9373_MOESM1_ESM.pdf]

Perceptions of Personal and Public Risk:

Dissociable effects on behavior and well-being

Laura K. Globig*, Bastien Blain & Tali Sharot*

Affective Brain Lab

Department of Experimental Psychology

& The Max Planck UCL Centre for Computational Psychiatry and Ageing Research

University College London, London, UK

*Correspondence to: laura.globig.15@ucl.ac.uk, t.sharot@ucl.ac.uk

Online Appendix

Supplementary Results.

Results: Time 1. Perceived relative personal risk, but not perceived public risk, is related to participants' happiness.

We asked our participants "Think about right now. How happy are you at this moment?". Responses were made on a continuous visual analogue scale ranging from 0 (very unhappy) to 100 (very happy)." In compliance with the policy of the Journal of Risk and Uncertainty ordinal scores were converted to 0/1. This was done as follows: scores below the midpoint of the scale (<50) were converted to 0 and those equal or above (>=50) were converted to 1. While perceived relative personal risk had a significant negative relationship with participants' happiness (Beta from a model predicting current happiness from perceived relative personal risk, perceived public risk and all demographic variables as controls revealed a significant effect for perceived relative personal risk β=-0.19, *p*=0.04) it was not related to perceived public risk (Beta from the same model β=-0.204, *p*=0.39, **Table S10**). Adding "sense of control" into our model revealed that sense of control was the variable most strongly associated with happiness (β=0.825, *p*<0.001, **Table S11**). These results replicate the findings described in the main test when using a slightly different question and scale to measure happiness.

Results: Time 2.

We had provided a summary in the main text of the results of Time 2. Below we provide additional detailed results.

Factors Predicting Perceived Relative Personal Risk and Perceived Public Risk.

Sense of control was inversely related to perceived relative personal risk (an ordinal logistic regression model predicting perceived relative personal risk from sense of control controlling for all demographic factors revealed a significant effect for sense of control; β=0.535, *p*=0.001, **Supplementary Figure 1a, Table S12**) and to perceived public risk measured by asking participants to estimate the risk to "a person" (a logistic regression model predicting perceived public risk from sense of control controlling for all demographic factors revealed a significant effect for sense of control: β=-0.59, *p*=0. 001, **Supplementary Figure 1b, Table S13**). A subset of the population in time 2 were also asked to estimate perceived danger to the health of the human population. This measure was not significantly related to sense of control possibly due to the smaller sample (Beta from a logistic model predicting estimated danger to the health of the human population from sense of control controlling for all demographic factors: β=-0.423, *p*=0.18, **Table S15**).

Democrats and females were more likely to perceive relative personal risk (Beta from a model including all demographic factors and sense of control: Political Orientation: β=-0.575, *p*<0.001, Gender: β=0.317, *p*=0.033) **Supplementary Figure 1a),** and perceived public risk (Betas from a model including all demographic factors and sense of control: Political Orientation: β=-0.74, *p*<0.001, Gender: β=0.558, *p*=0.001, **Supplementary Figure 1b**) as high. Older individuals were more likely to perceive relative personal risk as high (Age: β=0.017, *p*<0.001).

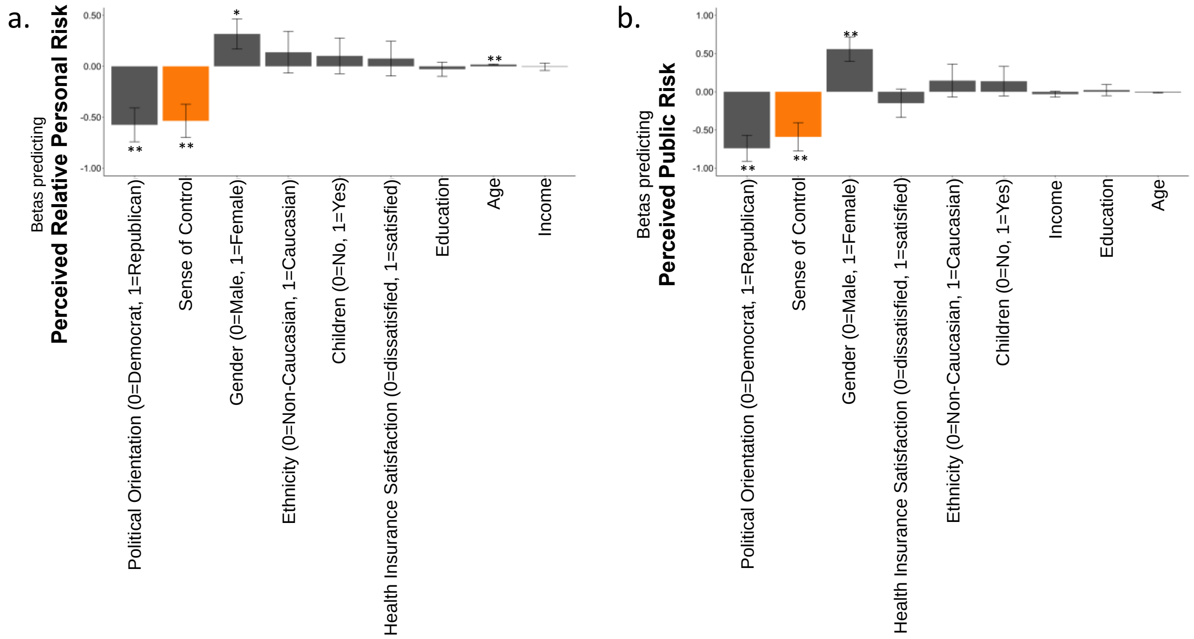

Supplementary Figure 1. High sense of control is associated with low risk perception (Time 2). Beta coefficients from two ordinal logistic regression model predicting (a) perceived relative personal risk (that is "Relative to others of your age and gender do you think you are less/more likely to get COVID-19?"and a logistic regression model predicting (b) perceived public risk (that is " How likely is a person to get COVID-19") in time 2. (a) People with strong sense of control (orange bar) were more likely to perceive relative personal risk as low, as were males, younger individuals, and Republicans. (b) People with strong sense of control (orange bar) were more likely to perceive public risk as low as were Republicans and males. Regressors are ordered from the largest magnitude to the smallest. **p*<0.05, ***p*<0.001, Error Bars SEM.

Perceived relative personal risk, but not perceived public risk, is related to participants' happiness.

As in time 1, perceived relative personal risk had a significant negative association with participants' happiness (Beta from a model predicting relative happiness from perceived relative personal risk, perceived public risk and all demographic variables as controls revealed a significant effect for perceived relative personal risk β=-0.298, *p*=0.011, **Supplementary Figure 2a & b, Table S16**) while perceived public risk did not (Beta from the same model β=-0.145 *p*=0.179, **Supplementary Figure 2a & c**).

Once again, adding "sense of control" into our model revealed that sense of control was the variable most strongly associated with happiness (β=1.59, p<0.001). Once again, this relationship was partially mediated by the sense of control (mediation analysis revealed indirect effect: β=-0.519, *p*=0.014, *Sobel Test: z*=-2.453). Once sense of control was statistically accounted for, the relationship between perceived relative personal risk and happiness was reduced to trend level (c': β=-0.225, *p*=0.071). The reverse mediation was not significant. That is perceived relative personal risk did not mediate the relationship between sense of control and happiness (indirect effect: β=-0.085, *p*=0.102, Sobel Test: z= 1.637).

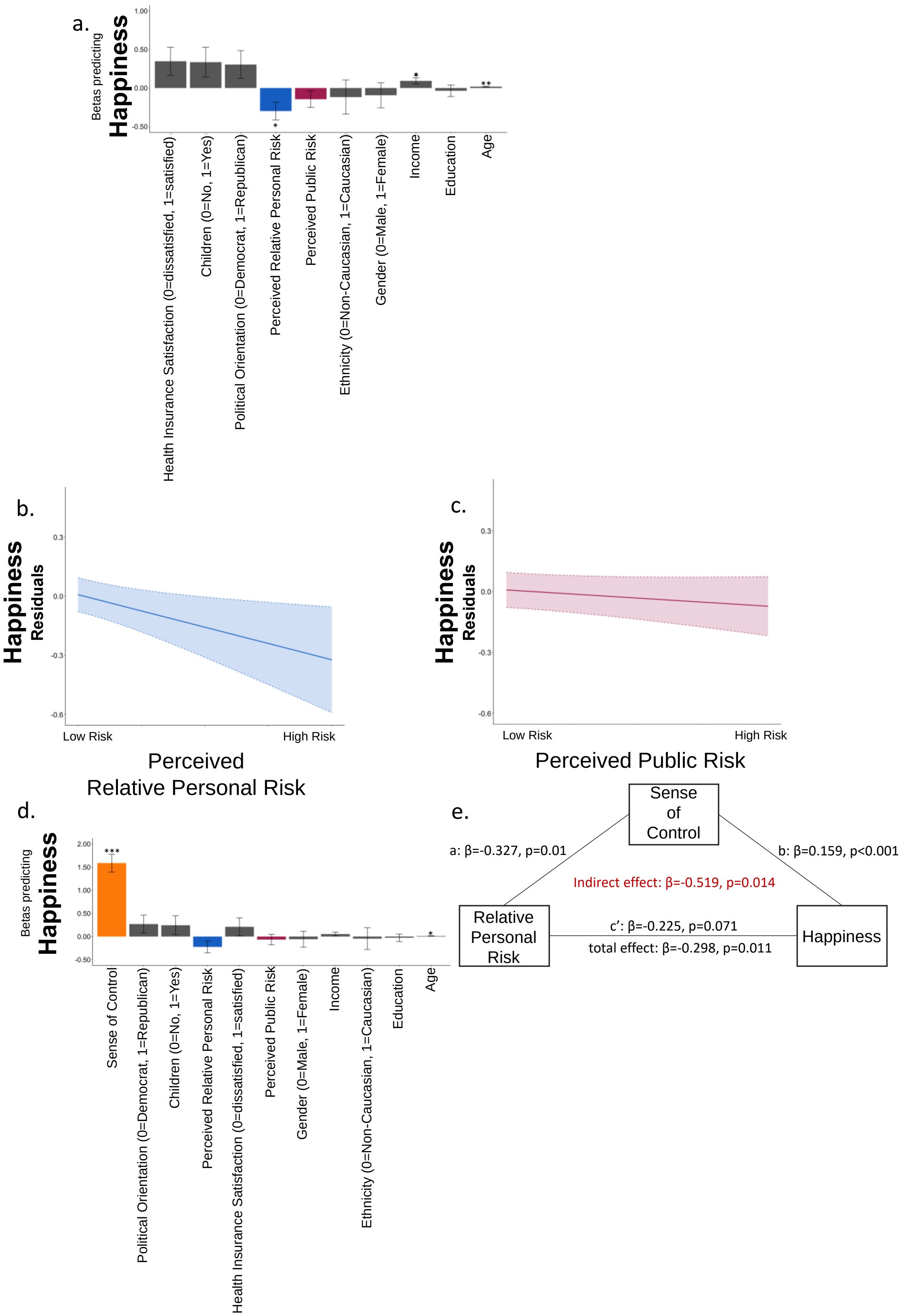

Figure 2. Perception of relative personal risk is related to happiness (time 2). (a) Displayed are the Beta coefficients from a logistic regression model predicting happiness, which shows that those who report low perceived relative personal risk (blue) are happier. Perceived public risk (red) is not

associated with happiness. These associations are also portrayed in **(b & c)**. Here, the Y and X axis display residuals from the same model, which includes all demographic controls. Clouds represent confidence intervals. **(d)** Adding sense of control (orange) to the model reveals that sense of control is the strongest factor predicting happiness and reduces the association between perceived relative personal risk and happiness. Indeed, a formal mediation model shows that **(e)** sense of control mediated the relationship between perceived relative personal risk and happiness. Regressors are ordered from the largest magnitude to the smallest. **p*< 0.05, **p<0.001, ***p<0.0001, Error Bars SEM.

Perceived relative personal risk and perceived public risk are related to anxiety.

As in time 1, both perceived relative personal risk and perceived public risk were strongly associated with high anxiety (Betas in a model including all demographics, perceived relative personal risk: $\beta=0.384$, $p=0.002$, **Supplementary Figure 3a & b**, perceived public risk: $\beta=0.249$, $p=0.022$, **Supplementary Figure 3a & c**, **Table S19**). When sense of control was added to the model, which in itself was negatively associated with anxiety and was the strongest predictor of anxiety ($\beta=-2.304$, $p<0.001$, **Supplementary Figure 3d**, **Table S20**), perceived relative personal risk was still a significant predictor of anxiety but was reduced in effect size ($\beta=0.31$, $p=0.021$) while perceived public risk was not ($\beta=0.163$, $p=0.172$). Both these relationships were mediated by a sense of control (partial mediation for relative personal risk: indirect effect: $\beta=0.753$, $p=0.014$, Sobel Test: $z=-2.468$, **Supplementary Figure 3e**; full mediation for perceived public risk: indirect effect: 0.685, $p=0.022$, Sobel Test: $z=2.298$, **Supplementary Figure 3f**). After accounting for a sense of control perceived personal risk was still related to anxiety (perceived relative personal risk; c': $\beta=0.31$, $p=0.021$) but perceived public risk was not (c': $\beta=0.163$, $p=0.172$). We did not find evidence for the reverse mediation. That is perceived risk did not mediate the relationship between sense of control and anxiety (indirect effect perceived personal risk: -0.044, $p=0.42$, Sobel Test: $z=0.803$; perceived public risk: 0.004, $p=0.88$, Sobel Test: $z=0.151$).

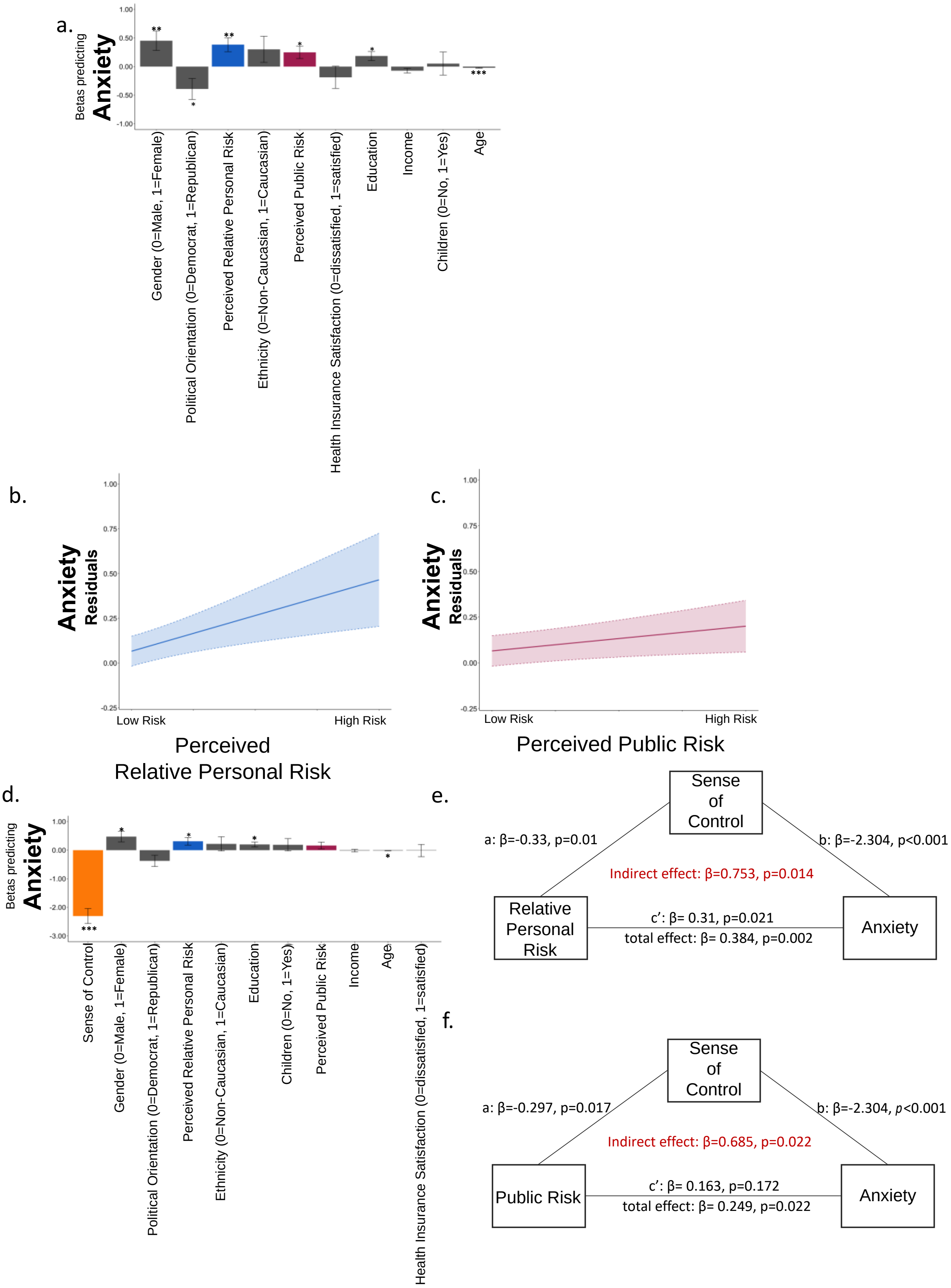

Supplementary Figure 3. Perception of risk to self and others is associated with anxiety (time 2). (a) Displayed are the Beta coefficients from a logistic regression model predicting anxiety (STAI scores), which shows that those who report higher perceived relative personal risk (blue) and perceived public risk (red) reported greater anxiety. These associations are also portrayed in **(b & c)**. Here, the Y and X axis display residuals from the same model, which includes all demographic controls. Clouds represent

confidence intervals. **(d)** Adding sense of control (orange) to the model reveals that sense of control is the strongest factor predicting anxiety and reduces the association between perceived relative personal risk and anxiety. Indeed, a formal mediation model shows that **(e)** sense of control partially mediated the relationship between perceived relative personal risk and anxiety and **(f)** fully mediated the relationship between public risk and anxiety. Regressors are ordered from the largest magnitude to the smallest. *$p<0.05$, **$p<0.001$, ***$p<0.0001$, Error Bars SEM.

Perceived public risk, but not perceived relative personal risk, is associated with behavioral compliance.

Behavioral compliance was high: 98.2% of participants reported putting effort into social distancing, 93% into frequent hand washing and 77.3% into avoidance of face touching, 96.8% reported they had not visit other people's homes in the last week and 87.7% reported they had not come within 1 meter of people outside their own residence. We found that while perceived public risk was strongly related with behavioral compliance (Beta in a model including perceived relative personal risk, perceived public risk and all demographic controls: $\beta=1.023$, $p=0.011$, **Supplementary Figure 5a & c, Table S22**) perceived relative personal risk was not ($\beta=-0.083$, $p=0.854$, **Supplementary Figure 5a & b**). Adding sense of control into the model did not alter the results (Sense of Control: $\beta=0.295$, $p=0.69$, **Supplementary Figure 5d, Table S23**). The relationship between perceived public risk and behavioral compliance could not be explained by high anxiety alone, as even when we add anxiety into the model the effect of perceived public risk on behavioral compliance remains significant (Betas in a model including perceived relative personal risk, perceived public risk, anxiety and all demographic controls Anxiety: $\beta=-0.238$, $p=0.716$, Perceived public risk: $\beta=0.105$, $p=0.01$, **Table S25**).

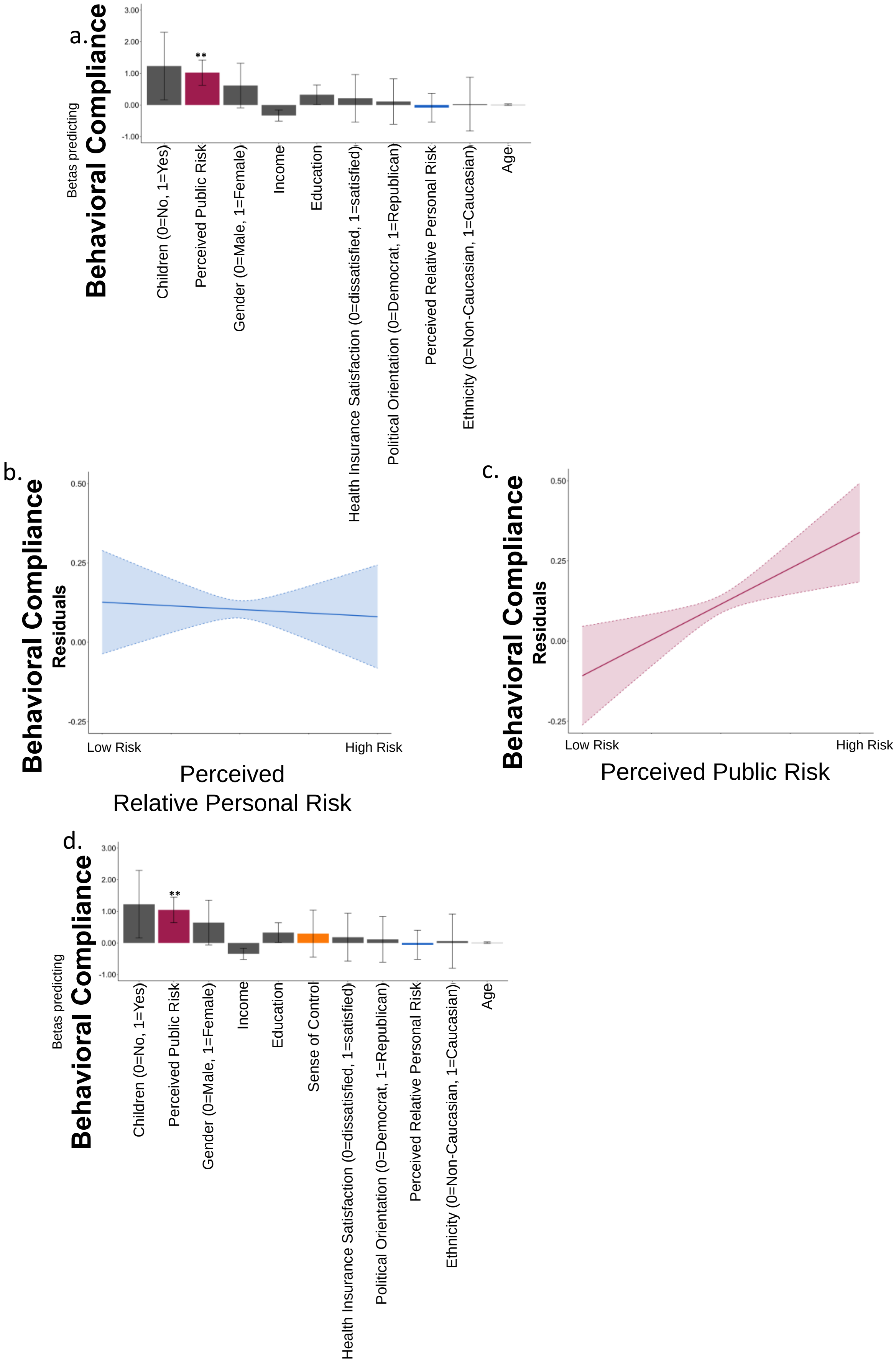
a.
Betas predicting
Behavioral Compliance
3.00
2.00
1.00
0.00
-1.00
**
Children (0=No, 1=Yes)
Perceived Public Risk
Gender (0=Male, 1=Female)
Income
Education
Health Insurance Satisfaction (0=dissatisfied, 1=satisfied)
Political Orientation (0=Democrat, 1=Republican)
Perceived Relative Personal Risk
Ethnicity (0=Non-Caucasian, 1=Caucasian)
Age
b.
Behavioral Compliance
Residuals
0.50
0.25
0.00
-0.25
Low Risk
High Risk
Perceived
Relative Personal Risk
c.
Behavioral Compliance
Residuals
0.50
0.25
0.00
-0.25
Low Risk
High Risk
Perceived Public Risk
d.
Betas predicting
Behavioral Compliance
3.00
2.00
1.00
0.00
-1.00
**
Children (0=No, 1=Yes)
Perceived Public Risk
Gender (0=Male, 1=Female)
Income
Education
Sense of Control
Health Insurance Satisfaction (0=dissatisfied, 1=satisfied)
Political Orientation (0=Democrat, 1=Republican)
Perceived Relative Personal Risk
Ethnicity (0=Non-Caucasian, 1=Caucasian)
Age

Supplementary Figure 4. Behavioral compliance is associated with perception of public risk, but not relative personal risk (time 2). (a) Displayed are the Beta coefficients from a logistic regression model predicting behavioral compliance, which shows that those who report higher perceived public risk (red) are more likely to comply. Perceived relative personal risk (blue), however, is not associated with behavioral compliance. These associations are also portrayed in **(b & c)**. Here, the Y and X axis display residuals from the same model, which includes all demographic controls. Clouds represent confidence intervals. **(d)** Adding sense of control (orange) to the model reveals that sense of control is not related to behavioral compliance. Regressors are ordered from the largest magnitude to the smallest. $^{**}p<0.001$, Error Bars SEM.

These results suggest that while perceived relative personal risk was related to happiness and anxiety, surprisingly it was not associated with behavioral compliance. Our results for time 2 thereby replicate those of time 1. Effects are thereby robust across times irrespective of phrasing of questions and time periods.

Regression Tables:
Time 1.

Table S1. Beta coefficients from a logistic regression predicting perceived personal risk at time 1.

Variable	Beta Estimate (SE)	p-value
Age	0.014 (0.004)	0.001
Gender (0=Male, 1=Female)	0.328 (0.121)	0.007
Children (0=No, 1=Yes)	0.063 (0.144)	0.661
Ethnicity (0=Non-Caucasian, 1=Caucasian)	0.257 (0.171)	0.134
Education	-0.072 (0.057)	0.210
Income	-0.032 (0.029)	0.279
Health Insurance Satisfaction (0=dissatisfied, 1=satisfied)	-0.053 (0.139)	0.703
Political Orientation (0=Democrat, 1=Republican)	-0.465 (0.135)	0.001
State Restrictions (0=low, 1=high)	0.039 (0.334)	0.907
Sense of Control	-0.277 (0.130)	0.033

Table S2. Beta coefficients from a logistic regression predicting perceived public risk at time 1.

Variable	Beta Estimate (SE)	p-value
Age	0.005 (0.008)	0.516
Gender (0=Male, 1=Female)	0.296 (0.230)	0.200
Children (0=No, 1=Yes)	-0.124 (0.267)	0.642
Ethnicity (0=Non-Caucasian, 1=Caucasian)	0.168 (0.318)	0.597
Education	0.013 (0.106)	0.906
Income	-0.003 (0.056)	0.956
Health Insurance Satisfaction (0=dissatisfied, 1=satisfied)	-0.104 (0.275)	0.705
Political Orientation (0=Democrat, 1=Republican)	-1.366 (0.237)	0.000
State Restrictions (0=low, 1=high)	-0.735 (0.754)	0.329
Sense of Control	-0.546 (0.269)	0.042

Table S3. Beta coefficients from a logistic regression predicting happiness at time 1, not including sense of control as predictor.

Variable	Beta Estimate (SE)	p-value

Age	0.012 (0.005)	0.009
Gender (0=Male, 1=Female)	-0.138 (0.133)	0.301
Children (0=No, 1=Yes)	0.047 (0.159)	0.766
Ethnicity (0=Non-Caucasian, 1=Caucasian)	0.321 (0.186)	0.084
Education	0.001 (0.063)	0.991
Income	0.097 (0.032)	0.003
Health Insurance Satisfaction (0=dissatisfied, 1=satisfied)	0.485 (0.153)	0.001
Political Orientation (0=Democrat, 1=Republican)	0.339 (0.149)	0.023
State Restrictions (0=low, 1=high)	-0.439 (0.375)	0.242
Perceived Relative Personal Risk	-0.201 (0.093)	0.030
Perceived Public Risk	0.036 (0.232)	0.877

Table S4. Beta coefficients from a logistic regression predicting happiness at time 1, including sense of control as predictor.

Variable	Beta Estimate (SE)	p-value
Age	0.011 (0.005)	0.000
Gender (0=Male, 1=Female)	-0.087 (0.136)	0.025
Children (0=No, 1=Yes)	0.066 (0.164)	0.522
Ethnicity (0=Non-Caucasian, 1=Caucasian)	0.324 (0.191)	0.686
Education	0.006 (0.064)	0.089
Income	0.084 (0.033)	0.925
Health Insurance Satisfaction (0=dissatisfied, 1=satisfied)	0.430 (0.156)	0.011
Political Orientation (0=Democrat, 1=Republican)	0.347 (0.152)	0.006
State Restrictions (0=low, 1=high)	-0.436 (0.384)	0.023
Perceived Relative Personal Risk	-0.167 (0.095)	0.256
Perceived Public Risk	0.136 (0.237)	0.080
Sense of Control	0.990 (0.147)	0.567

Table S5. Beta coefficients from a logistic regression predicting anxiety at time 1, not including sense of control as predictor.

Variable	Beta Estimate (SE)	p-value
Age	-0.027 (0.005)	0.000
Gender (0=Male, 1=Female)	0.538 (0.143)	0.000
Children (0=No, 1=Yes)	0.146 (0.177)	0.408
Ethnicity (0=Non-Caucasian, 1=Caucasian)	0.554 (0.197)	0.005
Education	0.039 (0.068)	0.565
Income	-0.060 (0.035)	0.085
Health Insurance Satisfaction (0=dissatisfied, 1=satisfied)	0.039 (0.167)	0.816
Political Orientation (0=Democrat, 1=Republican)	-0.377 (0.156)	0.015
State Restrictions (0=low, 1=high)	0.138 (0.391)	0.724
Perceived Relative Personal Risk	0.325 (0.101)	0.001
Perceived Public Risk	0.681 (0.235)	0.004

Table S6. Beta coefficients from a logistic regression predicting anxiety at time 1, including sense of control as predictor.

Variable	Beta Estimate (SE)	p-value
Age	-0.027 (0.005)	0.000

Gender (0=Male, 1=Female)	0.505 (0.146)	0.001
Children (0=No, 1=Yes)	0.128 (0.179)	0.475
Ethnicity (0=Non-Caucasian, 1=Caucasian)	0.582 (0.200)	0.004
Education	0.037 (0.069)	0.588
Income	-0.047 (0.035)	0.188
Health Insurance Satisfaction (0=dissatisfied, 1=satisfied)	0.102 (0.170)	0.550
Political Orientation (0=Democrat, 1=Republican)	-0.379 (0.158)	0.016
State Restrictions (0=low, 1=high)	0.129 (0.399)	0.747
Perceived Relative Personal Risk	0.302 (0.104)	0.003
Perceived Public Risk	0.622 (0.238)	0.009
Sense of Control	-0.854 (0.166)	0.000

Table S7. Beta coefficients from a logistic regression predicting behavioural compliance at time 1, not including sense of control as predictor.

Variable	Beta Estimate (SE)	p-value
Age	0.009 (0.015)	0.557
Gender (0=Male, 1=Female)	0.979 (0.469)	0.037
Children (0=No, 1=Yes)	-0.552 (0.469)	0.240
Ethnicity (0=Non-Caucasian, 1=Caucasian)	-0.512 (0.656)	0.435
Education	0.068 (0.201)	0.735
Income	0.013 (0.105)	0.904
Health Insurance Satisfaction (0=dissatisfied, 1=satisfied)	-0.257 (0.512)	0.616
Political Orientation (0=Democrat, 1=Republican)	0.583 (0.500)	0.244
State Restrictions (0=low, 1=high)	0.431 (1.072)	0.688
Perceived Relative Personal Risk	-0.272 (0.298)	0.362
Perceived Public Risk	2.026 (0.471)	0.000

Table S8. Beta coefficients from a logistic regression predicting behavioral compliance at time 1, including sense of control as predictor.

Variable	Beta Estimate (SE)	p-value
Age	0.009 (0.015)	0.561
Gender (0=Male, 1=Female)	0.920 (0.473)	0.052
Children (0=No, 1=Yes)	-0.571 (0.471)	0.225
Ethnicity (0=Non-Caucasian, 1=Caucasian)	-0.453 (0.658)	0.491
Education	0.066 (0.203)	0.746
Income	0.018 (0.106)	0.862
Health Insurance Satisfaction (0=dissatisfied, 1=satisfied)	-0.193 (0.514)	0.708
Political Orientation (0=Democrat, 1=Republican)	0.582 (0.500)	0.245
State Restrictions (0=low, 1=high)	0.419 (1.073)	0.697
Perceived Relative Personal Risk	-0.304 (0.303)	0.315
Perceived Public Risk	1.946 (0.476)	0.000
Sense of Control	-0.725 (0.569)	0.203

Table S9. Beta coefficients from a logistic regression predicting behavioral compliance at time 1 including anxiety as predictor but not sense of control.

Variable	Beta Estimate (SE)	p-value
Age	0.011 (0.016)	0.473

Gender (0=Male, 1=Female)	0.922 (0.473)	0.052
Children (0=No, 1=Yes)	-0.558 (0.471)	0.236
Ethnicity (0=Non-Caucasian, 1=Caucasian)	-0.524 (0.654)	0.423
Education	0.064 (0.202)	0.75
Income	0.019 (0.106)	0.857
Health Insurance Satisfaction (0=dissatisfied, 1=satisfied)	-0.269 (0.513)	0.6
Political Orientation (0=Democrat, 1=Republican)	0.617 (0.505)	0.222
State Restrictions (0=low, 1=high)	0.462 (1.070)	0.666
Perceived Relative Personal Risk	-0.317 (0.305)	0.298
Perceived Public Risk	1.980 (0.477)	0.00E+00
Anxiety	0.429 (0.450)	0.34

Table S10. Beta coefficients from a logistic regression predicting current happiness at time 1, not including sense of control as predictor.

Variable	Beta Estimate (SE)	p-value
Age	0.016 (0.005)	0.001
Gender (0=Male, 1=Female)	-0.119 (0.133)	0.372
Children (0=No, 1=Yes)	0.066 (0.160)	0.681
Ethnicity (0=Non-Caucasian, 1=Caucasian)	0.296 (0.182)	0.104
Education	-0.029 (0.062)	0.638
Income	0.108 (0.032)	0.001
Health Insurance Satisfaction (0=dissatisfied, 1=satisfied)	0.189 (0.150)	0.206
Political Orientation (0=Democrat, 1=Republican)	0.091 (0.150)	0.543
State Restrictions (0=low, 1=high)	-0.007 (0.370)	0.985
Perceived Relative Personal Risk	-0.190 (0.092)	0.040
Perceived Public Risk	-0.204 (0.237)	0.390

Table S11. Beta coefficients from a logistic regression predicting current happiness at time 1, including sense of control as predictor.

Variable	Beta Estimate (SE)	p-value
Age	0.015 (0.005)	0.002
Gender (0=Male, 1=Female)	-0.070 (0.136)	0.604
Children (0=No, 1=Yes)	0.082 (0.163)	0.613
Ethnicity (0=Non-Caucasian, 1=Caucasian)	0.295 (0.185)	0.111
Education	-0.024 (0.064)	0.701
Income	0.097 (0.033)	0.003
Health Insurance Satisfaction (0=dissatisfied, 1=satisfied)	0.128 (0.153)	0.403
Political Orientation (0=Democrat, 1=Republican)	0.087 (0.153)	0.569
State Restrictions (0=low, 1=high)	0.014 (0376)	0.970
Perceived Relative Personal Risk	-0.158 (0.094)	0.092
Perceived Public Risk	-0.127 (0.241)	0.597
Sense of Control	0.825 (0.141)	0.000

Time 2.

Table S12. Beta coefficients from a logistic regression predicting perceived relative personal risk at time 2, including sense of control as predictor.

Variable	Beta Estimate (SE)	p-value
Age	0.017 (0.005)	0.001
Gender (0=Male, 1=Female)	0.317 (0.149)	0.033
Children (0=No, 1=Yes)	0.138 (0.205)	0.500
Ethnicity (0=Non-Caucasian, 1=Caucasian)	0.103 (0.176)	0.558
Education	-0.029 (0.069)	0.674
Income	-0.005 (0.036)	0.897
Health Insurance Satisfaction (0=dissatisfied, 1=satisfied)	0.076 (0.171)	0.657
Political Orientation (0=Democrat, 1=Republican)	-0.575 (0.166)	0.001
Sense of Control	-0.535 (0.163)	0.001

Table S13. Beta coefficients from a logistic regression predicting perceived public risk at time 2, including sense of control as predictor.

Variable	Beta Estimate (SE)	p-value
Age	-0.010 (0.006)	0.091
Gender (0=Male, 1=Female)	0.558 (0.161)	0.001
Children (0=No, 1=Yes)	0.147 (0.216)	0.497
Ethnicity (0=Non-Caucasian, 1=Caucasian)	0.138 (0.194)	0.476
Education	0.022 (0.073)	0.763
Income	-0.031 (0.039)	0.426
Health Insurance Satisfaction (0=dissatisfied, 1=satisfied)	-0.150 (0.186)	0.422
Political Orientation (0=Democrat, 1=Republican)	-0.740 (0.170)	0.000
Sense of Control	-0.590 (0.185)	0.001

Table S14. Beta coefficients from a logistic regression predicting perceived absolute personal risk at time 2, including sense of control as predictor.

Variable	Beta Estimate (SE)	p-value
Age	0.007	0.160
Gender (0=Male, 1=Female)	0.430	0.003
Children (0=No, 1=Yes)	0.584	0.003
Ethnicity (0=Non-Caucasian, 1=Caucasian)	0.332	0.056
Education	0.111	0.104
Income	-0.006	0.878
Health Insurance Satisfaction (0=dissatisfied, 1=satisfied)	-0.160	0.341
Political Orientation (0=Democrat, 1=Republican)	-0.521	0.001
Sense of Control	-0.285	0.078

Table S15. Beta coefficients from a logistic regression predicting perceived danger to the health of the human population at time 2, including sense of control as predictor.

Variable	Beta Estimate (SE)	p-value
Age	0.019 (0.009)	0.049
Gender (0=Male, 1=Female)	0.143 (0.263)	0.587
Children (0=No, 1=Yes)	-0.354 (0.393)	0.368
Ethnicity (0=Non-Caucasian, 1=Caucasian)	0.082 (0.303)	0.787
Education	0.043 (0.118)	0.715
Income	-0.054 (0.065)	0.401
Health Insurance Satisfaction (0=dissatisfied, 1=satisfied)	-0.025 (0.308)	0.935

Political Orientation (0=Democrat, 1=Republican)	-1.675 (0.269)	0.000
Sense of Control	-0.423 (0.315)	0.180

Table S16. Beta coefficients from a logistic regression predicting happiness at time 2, not including sense of control as predictor.

Variable	Beta Estimate (SE)	p-value
Age	0.018 (0.006)	0.002
Gender (0=Male, 1=Female)	-0.093 (0.163)	0.566
Children (0=No, 1=Yes)	-0.118 (0.221)	0.593
Ethnicity (0=Non-Caucasian, 1=Caucasian)	0.336 (0.192)	0.080
Education	-0.035 (0.075)	0.639
Income	0.093 (0.039)	0.018
Health Insurance Satisfaction (0=dissatisfied, 1=satisfied)	0.348 (0.183)	0.057
Political Orientation (0=Democrat, 1=Republican)	0.305 (0.181)	0.091
Perceived Relative Personal Risk	-0.298 (0.117)	0.011
Perceived Public Risk	-0.145 (0.108)	0.179

Table S17. Beta coefficients from a logistic regression predicting happiness at time 2, including sense of control as predictor.

Variable	Beta Estimate (SE)	p-value
Age	0.013 (0.006)	0.037
Gender (0=Male, 1=Female)	-0.058 (0.173)	0.738
Children (0=No, 1=Yes)	-0.045 (0.234)	0.846
Ethnicity (0=Non-Caucasian, 1=Caucasian)	0.244 (0.203)	0.229
Education	-0.027 (0.080)	0.739
Income	0.055 (0.042)	0.189
Health Insurance Satisfaction (0=dissatisfied, 1=satisfied)	0.210 (0.195)	0.282
Political Orientation (0=Democrat, 1=Republican)	0.270 (0.191)	0.157
Perceived Relative Personal Risk	-0.225 (0.125)	0.072
Perceived Public Risk	-0.065 (0.114)	0.569
Sense of Control	1.586 (0.194)	0.000

Table S18. Beta coefficients from a logistic regression predicting happiness including absolute personal risk at time 2, not including sense of control as predictor.

Variable	Beta Estimate (SE)	p-value
Age	0.017 (0.006)	0.005
Gender (0=Male, 1=Female)	-0.099 (0.163)	0.543
Children (0=No, 1=Yes)	-0.092 (0.222)	0.680
Ethnicity (0=Non-Caucasian, 1=Caucasian)	0.347 (0.192)	0.071
Education	-0.025 (0.075)	0.744
Income	0.093 (0.039)	0.016
Health Insurance Satisfaction (0=dissatisfied, 1=satisfied)	0.333 (0.182)	0.067
Political Orientation (0=Democrat, 1=Republican)	0.328 (0.180)	0.067
Perceived Absolute Personal Risk	-0.149 (0.106)	0.159
Perceived Public Risk	-0.140 (0.155)	0.226

Table S19. Beta coefficients from a logistic regression predicting anxiety at time 2, not including sense of control as predictor.

Variable	Beta Estimate (SE)	p-value
Age	-0.021 (0.006)	0.001
Gender (0=Male, 1=Female)	0.452 (0.169)	0.007
Children (0=No, 1=Yes)	0.301 (0.228)	0.187
Ethnicity (0=Non-Caucasian, 1=Caucasian)	0.053 (0.201)	0.792
Education	0.185 (0.079)	0.019
Income	-0.071 (0.041)	0.081
Health Insurance Satisfaction (0=dissatisfied, 1=satisfied)	-0.187 (0.194)	0.336
Political Orientation (0=Democrat, 1=Republican)	-0.391 (0.183)	0.033
Perceived Relative Personal Risk	0.384 (0.122)	0.002
Perceived Public Risk	0.249 (0.109)	0.022

Table S20. Beta coefficients from a logistic regression predicting anxiety including sense of control at time 2.

Variable	Beta Estimate (SE)	p-value
Age	-0.015 (0.007)	0.023
Gender (0=Male, 1=Female)	0.473 (0.184)	0.010
Children (0=No, 1=Yes)	0.220 (0.249)	0.377
Ethnicity (0=Non-Caucasian, 1=Caucasian)	0.188 (0.215)	0.383
Education	0.200 (0.086)	0.020
Income	-0.017 (0.045)	0.699
Health Insurance Satisfaction (0=dissatisfied, 1=satisfied)	-0.015 (0.214)	0.946
Political Orientation (0=Democrat, 1=Republican)	-0.374 (0.198)	0.059
Perceived Relative Personal Risk	0.310 (0.134)	0.021
Perceived Public Risk	0.163 (0.119)	0.172
Sense of Control	-2.304 (0.262)	0.001

Table S21. Beta coefficients from a logistic regression predicting anxiety including absolute personal risk at time 2, not including sense of control as predictor.

Variable	Beta Estimate (SE)	p-value
Age	-0.019 (0.006)	0.002
Gender (0=Male, 1=Female)	0.457 (0.168)	0.007
Children (0=No, 1=Yes)	0.265 (0.229)	0.247
Ethnicity (0=Non-Caucasian, 1=Caucasian)	0.042 (0.201)	0.835
Education	0.169 (0.079)	0.031
Income	-0.072 (0.041)	0.075
Health Insurance Satisfaction (0=dissatisfied, 1=satisfied)	-0.169 (0.193)	0.382
Political Orientation (0=Democrat, 1=Republican)	-0.422 (0.182)	0.020
Perceived Absolute Personal Risk	0.204 (0.110)	0.064
Perceived Public Risk	0.237 (0.116)	0.041

Table S22. Beta coefficients from a logistic regression predicting behavioural compliance at time 2, including sense of control as predictor.

Variable	Beta Estimate (SE)	p-value
Age	0.011 (0.023)	0.644

Gender (0=Male, 1=Female)	0.615 (0.705)	0.383
Children (0=No, 1=Yes)	0.026 (0.851)	0.976
Ethnicity (0=Non-Caucasian, 1=Caucasian)	1.230 (1.070)	0.250
Education	0.324 (0.311)	0.296
Income	-0.333 (0.176)	0.058
Health Insurance Satisfaction (0=dissatisfied, 1=satisfied)	0.212 (0.752)	0.778
Political Orientation (0=Democrat, 1=Republican)	0.110 (0.717)	0.878
Perceived Relative Personal Risk	-0.084 (0.454)	0.854
Perceived Public Risk	1.023 (0.400)	0.010

Table S23. Beta coefficients from a logistic regression predicting behavioral compliance at time 2, including sense of control as predictor.

Variable	Beta Estimate (SE)	p-value
Age	0.010 (0.023)	0.677
Gender (0=Male, 1=Female)	0.646 (0.709)	0.362
Children (0=No, 1=Yes)	0.058 (0.857)	0.946
Ethnicity (0=Non-Caucasian, 1=Caucasian)	1.223 (1.069)	0.253
Education	0.328 (0.311)	0.292
Income	-0.344 (0.177)	0.053
Health Insurance Satisfaction (0=dissatisfied, 1=satisfied)	0.181 (0.757)	0.811
Political Orientation (0=Democrat, 1=Republican)	0.114 (0.721)	0.874
Perceived Relative Personal Risk	-0.059 (0.458)	0.898
Perceived Public Risk	1.046 (0.404)	0.010
Sense of Control	0.295 (0.740)	0.691

Table S24. Beta coefficients from a logistic regression predicting behavioural compliance at time 2, including absolute personal risk as predictor but not sense of control as predictor.

Variable	Beta Estimate (SE)	p-value
Age	0.012 (0.023)	0.593
Gender (0=Male, 1=Female)	0.645 (0.710)	0.364
Children (0=No, 1=Yes)	0.091 (0.863)	0.916
Ethnicity (0=Non-Caucasian, 1=Caucasian)	1.239 (1.070)	0.247
Education	0.364 (0.316)	0.249
Income	-0.327 (0.176)	0.063
Health Insurance Satisfaction (0=dissatisfied, 1=satisfied)	0.200 (0.755)	0.791
Political Orientation (0=Democrat, 1=Republican)	0.087 (0.714)	0.904
Perceived Absolute Personal Risk	-0.519 (0.404)	0.198
Perceived Public Risk	1.208 (0.422)	0.004

Table S25. Beta coefficients from a logistic regression predicting behavioural compliance at time 2, including anxiety as predictor but not sense of control.

Variable	Beta Estimate (SE)	p-value
Age	0.010 (0.023)	0.677
Gender (0=Male, 1=Female)	0.653 (0.712)	0.359
Children (0=No, 1=Yes)	0.026 (0.855)	0.976
Ethnicity (0=Non-Caucasian, 1=Caucasian)	1.229 (0.1069)	0.250
Education	0.339 (0.314)	0.281
Income	-0.340 (0.177)	0.054

Health Insurance Satisfaction (0=dissatisfied, 1=satisfied)	0.231 (0.752)	0.758
Political Orientation (0=Democrat, 1=Republican)	0.100 (0.715)	0.889
Perceived Absolute Personal Risk	-0.077 (0.454)	0.865
Perceived Public Risk	1.051 (0.408)	0.010
	-0.238 (0.655)	0.716

Supplementary Methods

Additional information obtained in questionnaire (which are part of parallel studies):

Behavioral Change. To assess behavioral change participants were asked to indicate the frequency of face-to-face interaction, online/telephone interaction, physical activity, outdoor activity, visiting places of religious worship before and after the restrictions.
Addictive behaviors. Participants reported frequency of habitudinal and addictive behaviors including smoking, alcohol consumption, gambling, eating before and after the restrictions.
Psychopathology. Participants completed the Obsessive-Compulsive Inventory – Revised (OCI-R, Foa, Kozak, Salkovskis, Coles, & Amir, 1998), Patient Health Questionnaire (PHQ-9, Kroenke, Spitzer, & Williams, 2001), Apathy Evaluation Scale (AES, Mann, 1990).
Psychosocial Questionnaires: Participants completed a series of psychosocial questionnaires assessing empathic concern (Davis, 1983), resilience (Smith et al., 2008), narcissism (Leckelt et al., 2018), risk-taking propensity (GRiPS, Zhang, Highhouse, & Nye, 2019).
Stress Coping. Participants responded on a 5-point Likert Scale from 1 (strongly disagree) to 5 (strongly agree) to the following items: 1) The effects of stress are negative and should be avoided. 2) The effects of stress are positive and should be utilized.
Health Anxiety. Participants were asked to indicate health anxiety on a 5-point Likert Scale from 1 (very inaccurate) to 5 (very accurate): Often I am concerned about diseases I might have.
Non-Conformity. Participants were asked to indicate conformity on a 5-point Likert Scale from 1 (very strong disagreement) to 5 (very strong agreement): I prefer to make my own way in life rather than find and follow.
Social Support and Connectedness. Participants were asked to indicate on a 7-point Likert Scale from 1 (strongly disagree) to 7 (strongly agree): My friends/family give me the support I need. We presented participants with a modified version of the "inclusion of others in the self" scale (Aron, Aron, Tudor, & Nelson, 1991).
Behavioral Economic Tasks. Participants completed a series of established behavioral tasks including a one-shot dictator game (Kahneman, 2016), an intertemporal choice task (Kirby & Maraković, 1996) and a loss aversion task (Rutledge, Skandali, Dayan, & Dolan, 2014).
Anxiety about possible implications of COVID-19. Participants indicated how anxious they were on a scale of 0 (not at all) to 100 (very much): a) "Are you anxious about your own health in light of COVID-19?"; b) "Are you anxious about the health of your loved ones in light of COVID-19?"; c) "Are you anxious about dealing with lockdown in your area?"; d) "Are you anxious about the consequence to your income/savings in light of COVID-19?"; e) "Are you anxious about homeschooling in light of COVID-19?"; f) "Are you anxious about not being able to exercise?"; g) "Are you anxious about not having access to food/medicine/other supplies?"; h) "Are you anxious about not being able to socialize?"
LOT-R. Participants also completed the Life Orientation Optimism Test (LOT-R, Molina et al., 2013) that measures trait optimism.

References

Aron, A., Aron, E. N., Tudor, M., & Nelson, G. (1991). Close relationships as including other in the self. *Journal of Personality and Social Psychology*, *60*(2), 241–253. https://doi.org/10.1037/0022-3514.60.2.241

Davis, M. H. (1983). A Mulitdimensional Approach to Individual Differences in Empathy. *Journal of Personality and Social Psychology*, *44*(1), 113–126. https://doi.org/10.1037/0022-3514.44.1.113

Foa, E. B., Kozak, M. J., Salkovskis, P. M., Coles, M. E., & Amir, N. (1998). The validation of a new obsessive-compulsive disorder scale: The obsessive-compulsive inventory. *Psychological Assessment*, *10*(3), 206–214. https://doi.org/10.1037/1040-3590.10.3.206

Kahneman, D. (2016). *Fairness and the Assumptions of Economics Author (s): Daniel Kahneman , Jack L . Knetsch and Richard H . Thaler Source : The Journal of Business , Vol . 59 , No . 4 , Part 2 : The Behavioral Foundations of Economic Published by : The University of Chica*. *59*(4).

Kirby, K. N., & Maraković, N. N. (1996). Delay-discounting probabilistic rewards: Rates decrease as amounts increase. *Psychonomic Bulletin and Review*, *3*(1), 100–104. https://doi.org/10.3758/BF03210748

Kroenke, K., Spitzer, R. L., & Williams, J. B. W. (2001). The PHQ-9: Validity of a brief depression severity measure. *Journal of General Internal Medicine*, *16*(9), 606–613. https://doi.org/10.1046/j.1525-1497.2001.016009606.x

Leckelt, M., Wetzel, E., Gerlach, T. M., Ackerman, R. A., Miller, J. D., Chopik, W. J., … Back, M. D. (2018). Validation of the narcissistic admiration and rivalry questionnaire short scale (NARQ-S) in convenience and representative samples. *Psychological Assessment*, *30*(1), 86–96. https://doi.org/10.1037/pas0000433

Mann, R. S. (1990). Differential diagnosis and classification of apathy. *Am J Psychiatry*, *147*(1), 22–30.

Molina, K. M., Molina, K. M., Goltz, H. H., Kowalkouski, M. A., Hart, S. L., Latini, D., … Gidron, Y. (2013). Revised Life Orientation Test (LOT-R). *Encyclopedia of Behavioral Medicine*, 1678–1678. https://doi.org/10.1007/978-1-4419-1005-9_101490

Rutledge, R. B., Skandali, N., Dayan, P., & Dolan, R. J. (2014). A computational and neural model of momentary subjective well-being. *Proceedings of the National Academy of Sciences of the United States of America*, *111*(33), 12252–12257. https://doi.org/10.1073/pnas.1407535111

Smith, B. W., Dalen, J., Wiggins, K., Tooley, E., Christopher, P., & Bernard, J. (2008). The brief resilience scale: Assessing the ability to bounce back. *International Journal of Behavioral Medicine*, *15*(3), 194–200. https://doi.org/10.1080/10705500802222972

Zhang, D. C., Highhouse, S., & Nye, C. D. (2019). Development and validation of the General Risk Propensity Scale (GRiPS). *Journal of Behavioral Decision Making*, *32*(2), 152–167. https://doi.org/10.1002/bdm.2102
